# Exosomal Signaling during Hypoxia Mediates Microvascular Endothelial Cell Migration and Vasculogenesis

**DOI:** 10.1371/journal.pone.0068451

**Published:** 2013-07-08

**Authors:** Carlos Salomon, Jennifer Ryan, Luis Sobrevia, Miharu Kobayashi, Keith Ashman, Murray Mitchell, Gregory E. Rice

**Affiliations:** 1 University of Queensland Centre for Clinical Research, Herston, Queensland, Australia; 2 Cellular and Molecular Physiology Laboratory (CMPL), Division of Obstetrics and Gynaecology, School of Medicine, Faculty of Medicine, Pontificia Universidad Católica de Chile, Santiago, Chile; University of Bristol, United Kingdom

## Abstract

Vasculogenesis and angiogenesis are critical processes in fetal circulation and placental vasculature development. Placental mesenchymal stem cells (pMSC) are known to release paracrine factors (some of which are contained within exosomes) that promote angiogenesis and cell migration. The aims of this study were: to determine the effects of oxygen tension on the release of exosomes from pMSC; and to establish the effects of pMSC-derived exosomes on the migration and angiogenic tube formation of placental microvascular endothelial cells (hPMEC). pMSC were isolated from placental villi (8–12 weeks of gestation, n = 6) and cultured under an atmosphere of 1%, 3% or 8% O_2_. Cell-conditioned media were collected and exosomes (exo-pMSC) isolated by differential and buoyant density centrifugation. The dose effect (5–20 µg exosomal protein/ml) of pMSC-derived exosomes on hPMEC migration and tube formation were established using a real-time, live-cell imaging system (Incucyte™). The exosome pellet was resuspended in PBS and protein content was established by mass spectrometry (MS). Protein function and canonical pathways were identified using the PANTHER program and Ingenuity Pathway Analysis, respectively. Exo-pMSC were identified, by electron microscopy, as spherical vesicles, with a typical cup-shape and diameters around of 100 nm and positive for exosome markers: CD63, CD9 and CD81. Under hypoxic conditions (1% and 3% O_2_) exo-pMSC released increased by 3.3 and 6.7 folds, respectively, when compared to the controls (8% O_2_; *p*<0.01). Exo-pMSC increased hPMEC migration by 1.6 fold compared to the control (*p*<0.05) and increased hPMEC tube formation by 7.2 fold (*p*<0.05). MS analysis identified 390 different proteins involved in cytoskeleton organization, development, immunomodulatory, and cell-to-cell communication. The data obtained support the hypothesis that pMSC-derived exosomes may contribute to placental vascular adaptation to low oxygen tension under both physiological and pathological conditions.

## Introduction

Exosomes are secreted nanovesicles (30–100 nm diameter) formed through the inward budding of multivesicular bodies (MVBs) that traffic and transfect proteins, mRNAs and miRNAs into target cells [1]. The significance of exosomal signaling in diverse aspects of physiology and pathophysiology has only recently been recognized [2]. Exosomes have now been reported to display immunomodulatory activity [3], [4] containing molecules such as HLA-G5, B7–H1, B7–H3 [5] and syncytian-1 [6] from trophoblast cells, suppression and activation of natural killer cells and macrophages [7], [8]; promote cell migration and metastasis [9], [10], traffic hydrophobic mediators of cell differentiation [11] and viral proteins [12]; and promote allograft survival and induce donor specific allograft tolerance [13]. Of particular relevance to this study, exosomes released from progenitor cells stimulate: endothelial cell migration [14]; tissue vascularization and angiogenesis [15], [16]; induce cell proliferation [17]; and are cardioprotective of ischemia/reperfusion injury [18].

Mesenchymal stem cells are archetypal multipotent progenitor cells that display fibroblastic morphology and plasticity to differentiate into diverse cell types including: osteocytes, adipocytes and endothelial cells. MSCs are isolated from various sources including bone marrow (principal source), adipose tissue and placenta. Within the human placenta, MSC have been isolated from umbilical cord blood and chorionic villi [19], [20] displaying phenotypes comparable to those isolated from bone marrow, including surface antigen expression (CD45^−^, CD14^−^, CD19^−^, CD80^+^, CD86^+^, CD40^+^ and B7H2^+^) and the capacity to differentiate into multiple linages *in vitro*. MSC have been implicated in wound healing and display the ability to migrate to sites of injury and engage in tissue repair and regeneration of bone, cartilage, liver tissue or myocardial cells [21], [22]. MSC modulate immune responses in collagen disease, multiple sclerosis and transplants bone marrow and contribute to vasculogenesis, angiogenesis and endothelial repair [23], processes that are fundamental for tissue repair. MSC affect tissue repair through the release of paracrine mediators [24]–[27] including exosomes [28].

MSCs are present in the first trimester human placenta, however, their role in placental vascular development remains to be established. During early pregnancy, the placental vasculature develops under hypoxic conditions. During the first trimester, placental PO_2_ is ∼ 2.6% before placento-maternal perfusion is established. At around 12 weeks of pregnancy, the placenta is perfused with maternal blood and PO_2_ increases to ∼ 8% [29], [30]. There is a paucity of information about the role of MSC and, in particular, the release of exosomes from MSC during this critical period of vascular development. Of note, however, Hofmann *et al.,* [31], recently proposed that exosomes may function as part of an oxygen sensing mechanism that promotes vasculogenesis and angiogenesis. We hypothesize that: (i) exosomes released by pMSC act paracellularly to promote cell migration and angiogenesis within the placental villus tree; and (ii) that the release of exosomes from pMSC is responsive to changes in oxygen tension.

The aim of this study, therefore, was to establish the effect of oxygen tension on the release of exosomes from pMSC; and the effects of pMSC-derived exosomes on the migration and angiogenic tube formation of human placental microvascular endothelial cells (hPMEC). pMSC-derived exosomes promote hPMEC cell migration and tube formation *in vitro.* The release and bioactivity of pMSC-derived exosomes is oxygen tension dependent. The data obtained are consistent with the hypothesis that pMSC-derived exosomes are released under hypoxic conditions and promote angiogenesis within the developing placenta.

## Materials and Methods

### First Trimester and Term Placental Collection

Tissue collection was approved by the Human Research Ethics Committees of the Royal Brisbane and Women’s Hospital, and the University of Queensland (HREC/09/QRBW/14). All experiments and data collection and analyses were conducted with an ISO 17025 and 21 CFR part 11 conforming laboratory environment. Written informed consent was obtained from women for the use of placental tissue for research purposes after clinically indicated termination of pregnancy in compliance with national research guidelines.

### Isolation of Placental Mesenchymal Stem Cells

pMSC were isolated, from placental villi by enzymatic digestion using protocols adapted from *Steigman & Fauza* [32]. Briefly, placental chorionic villi (n = 6; 8–12 weeks gestational age) were separated from the remainder of the placenta unit and were washed in PBS. The villi were minced into small pieces and were transferred in to 50 ml tubes. The tissues were enzymatic digestion with dispase (2.4 U/ml) and collagenase (240 U/ml) made in PBS. The tissues were digested for 1 hr at 37°C on a rocker. The single cell suspension was then filtered through a 100 µm mesh into a new tube. The cells were centrifuged for 15 mins at 500×g at RT and the pellet was resuspended in 10 ml cDMEM. pMSC were cultured in Dulbecco’s modified Eagle’s medium (DMEM) (Life Technologies™, Carlsbad, CA), supplemented with 10% fetal bovine serum, 100 IU/mL penicillin, and 100 µg/mL streptomycin (Life Technologies™), at 37°C with 5% CO2. pMSC were characterised by well-established cell surface markers. All cells used in this study were passaged less than 6.

### MSC Differentiation Assays

The differentiation potential of placental villi MSC was established according to previously published methods [33]. For adipogenesis, 2×10^5^ pMSC were seeded in 6 well plates until confluent and differentiation was induced by indomethacin (60 µM) dexamethasone (1 µm), insulin (5 µg/ml) and isobutylmethylxanthine (IBMX) (0.5 mM). After 21 days, cells were fixed with 10% formalin and stained with Oil Red O. Adipogenic differentiation was determined by the appearance of Oil Red O. Osteogenic differentiation was induced by culturing 3×10^5^ cells in 6 well plates in the presence of osteogenic induction media containing dexamethasone (0.1 µM), β-glycerol phosphate (10 mM) and L-ascorbate-2-phosphate (0.2 mM) for 21 days. Cells were fixed in 10% formalin and stained with Alizarin red. Differentiation was determined by the appearance of red deposits, representing areas of mineralized calcium. All reagents were from Sigma-Aldrich. Staining for both adipogenic and osteogenic assays was visualized using bright field phase contrast microscopy.

### Isolation of hPMEC

The effects of exosomes on endothelial cell migration and angiogenesis were assessed using human placenta microvascular endothelial cells (hPMEC). hPMEC were isolated as previously described [34]. In brief, chorionic villi obtained from placental tissue samples (∼4 cm^3^ of the chorionic villous) were digested with trypsin/EDTA (0.25/0.2%, 20 min, 37°C) followed by 0.1 mg/ml collagenase (2 h, 37°C, Type II Clostridium histolyticum; Boehringer, Mannheim, Germany) in medium 199 (M199, Gibco Life Technologies, Carlsbad, CA, USA) Digested tissue was resuspended in M199 containing 5 mM D-glucose, 20% newborn calf serum (NBCS), 20% fetal calf serum (FCS), 3.2 mM L-glutamine and 100 U/ml penicillin streptomycin (primary culture medium, PCM), and filtered through a 55 µm pore size Nylon mesh. Filtered cell suspension was transferred into a 1% gelatin-coated T25 culture flask for culture (37°C, 5% O_2_, 5% CO_2_) in PCM. After 5 days, confluent cells were trypsinized (trypsin/EDTA 0.25/0.2%, 3 min, 37°C) and subjected to CD31 (against platelet endothelial cell adhesion molecule 1, PECAM-1)-positive immunoselection using Dynabeads CD31 microbeads from MACS® (Miltenyi Biotech, Bergisch-Gladbach, Germany). Endothelial cells immunoselection was performed mixing anti-CD31 antibody-magnetic coated microbeads with the cell suspension (48×10^3^ beads/ml cell suspension, 20 min, 4°C). Suspension medium was discarded and cells attached to the magnetic microbeads were collected and washed (3 times) in HBSS (37°C). CD31-coated microbead-attached cells were resuspended in PCM containing 10% NBCS and 10% FCS, and cultured under standard conditions (37°C, 5% CO2) until confluence in passage 3. Immunocytochemistry analysis established that more than 96% of cells in the endothelial preparations used in the present study, were positive for von Willebland Factor (vWF) and CD31 (data not shown).

### Flow Cytometry

The expression of cell surface and intracellular antigens was assessed by flow cytometry (FACScalibur™, Becton Dickinson, San Jose, CA, USA). To identify intra-cellular antigens, cells were detached, blocked with 1% bovine serum albumin (BSA; Sigma, St. Louis, MO) in phosphate buffered saline (PBS, Life Technologies™) then fixed with 0.01% paraformaldehyde (PFA) (Sigma) and permeabilized with 0.5% Triton X-100. To characterize the expression of cell surface and intracellular antigens, cells were detached and blocked with 1% BSA and incubated with specific anti-human primary antibodies, either conjugated with PE, FITC or PE-Cy5 or unconjugated. For unconjugated antibodies, cells were subsequently washed with 1% BSA and incubated with secondary goat anti-murine IgM PE (Santa Cruz Biotechnology®, Santa Cruz, CA, USA). All samples were analyzed in triplicate by FACScalibur™ flow cytometry (Becton Dickinson). Positive controls were hESC and negative controls were IgG or IgM primary antibody-specific isotype controls.

### Hypoxia

The effects of oxygen tension on the release of exosomes from pMSC were assessed by incubating cells for 48 h (in exosome-free culture medium) under an atmosphere of 5% CO_2_-balanced N_2_ to obtain 1%, 3% or 8% O_2_ (pO_2_ ∼6.75, ∼20.25 or ∼54 mmHg, respectively) in an automated PROOX 110-scaled hypoxia chamber (BioSpherics™, Lacona, NY, USA). Cell number and viability was determined after each experimental treatment by Trypan Blue exclusion and Countess® Automated cell counter (Life Technologies™). Proliferation data was collected for all the experimental conditions and in particular to assess the effects of proliferation hypoxic conditions using a real-time cell imaging system (IncuCyte™ live-cell ESSEN BioScience Inc, Ann Arbor, Michigan, USA). In all experiments, viability remained at >95%. Incubation media pO_2_ and pH were independently confirmed using a blood gas analyzer (Radiometer®, Brønshøj, Denmark) and NeoFox oxygen probe (Ocean Optics ™, Dunedin, FL, USA). HIF expression was used in Western blot analysis as a positive control for hypoxia in MSC (data not show).

### Isolation and Purification of pMSC Exosomes

Exosomes were isolated from cell-free pMSC as previously described [35]. In brief, pMSC-conditioned media was centrifuged at 300×g for 15 min, 2000×g for 30 min, and 12000×g for 45 min to remove whole cells and debris. The resultant supernatant were passed through a 0.22 µm filter sterilize Steritop™ (Millipore, Billerica, MA, USA) and then centrifuged at 100,000×g for 75 min (Thermo Fisher Scientific Ins., Asheville, NC, USA, Sorvall, SureSpin™ 630/36, fixed angle rotor). The pellet was resuspended in PBS, washed and re-centrifuged (100,000×g, 75 min). The pellet was resuspended in PBS, layered on a cushion of 30% (w/v) sucrose and centrifuged at 110,000 g for 75 min. The fraction containing pMSC exosomes (∼3.5 ml, 1.1270 density using OPTi digital refractometer (Bellingham^+^Stanley Inc., Lawrenceville, GA, USA) was recovered with an 18-G needle and diluted in PBS, and then ultracentrifuged at 110 000×g of 70 min. Recovered exosomes were resuspended in 50 µl PBS and their protein contents were determined using the Bradford assay (Bio-Rad DC) [35]. Exosome samples (5 µl) were prepared by adding RIPA buffer (50 mM Tris, 1% Triton×100, 0.1% SDS, 0.5% DOC, 1 mM EDTA, 150 mM NaCl, protease inhibitor) directly to exosomes suspended in PBS and sonicated at 37°C for 15 s three times to lyse exosome membrane and solubilise the proteins. Bovine serum albumin (BSA) diluted in RIPA buffer and PBS mixture (1∶1) were prepared as protein standards (0, 200, 400, 600, 800, 1000, 1500 µg/mL). Standards and samples (exosomes) were transferred to 96-well plates and procedures outlined by the manufacture were followed. In brief, alkaline copper tartrate solution (BIO-RAD, USA) and dilute Folin Reagent (BIO-RAD, USA) were added to the samples and incubated for 15 min. The absorbance was read at 750 *nm* with Paradigm Detection Platform (Beckman Coulter, USA).

### Transmission Electron Microscopy

The exosome fraction isolated by differential and buoyant density gradient centrifugation was assessed by transmission electron microscopy. Exosome pellets (as described above) were fixed in 3% (w/v) glutaraldehyde and 2% paraformaldehyde in cacodylate buffer, pH 7.3. Five microlitres of sample was then applied to a continuous carbon grid and negatively stained with 2% uranyl acetate. The samples were examined in an FEI Tecnai 12 transmission electron microscope (FEI™, Hillsboro, Oregon, USA).

### Western Blot

Exosome proteins separated by polyacrylamide gel electrophoresis were transferred to Immobilon-®FL polyvinylidene difluoride membranes (Millipore, Billerica, MA, USA) and probed with primary mouse monoclonal anti-CD63 (1∶2000), anti-CD81 (1∶1500) or anti-CD9 (1∶1500) as previously described [35] for specific exosome markers. Membranes were washed in Tris buffer saline Tween, and incubated (1 h) in TBST/0.2% BSA containing horseradish peroxidase-conjugated goat anti-mouse antibody. Proteins were detected by enhanced chemiluminescence with the SRX-101A Tabletop Processor (Konica Minolta, Ramsey, NJ, USA). The relative intensity of the bands was determined by densitometry using the GS-800 Calibrated Densitometer (Bio-Rad Laboratories, Hercules, CA, USA).

### Migration and Tube Formation Assay

To assess the effect of exosomes on endothelial cell tube formation, hPMEC were cultured in 96 or 48-well culture plates (Corning Life Science, Tewksbury, MA, USA) according to the manufacturer’s instructions and visualized using a real-time cell imaging system (IncuCyte™ live-cell ESSEN BioScience Inc, Ann Arbor, Michigan, USA). Cells were imaged every hour to monitor treatment-induced cell migration, tube formation, confluence and morphologic changes. Cell migration was assessed by scratch assays, in which, hPMEC were grown to confluence and then a scratch was made using a 96-pin WoundMaker™. The wells were washed with PBS to remove any debris and incubated in the presence of 0 (control) 5, 10 or 20 µg protein/ml of pMSC-derived exosome isolated from cells cultured under 1%, 3% or 8% O_2_. Wound images were automatically acquired and registered by the IncuCyte™ software system. Typical kinetic updates were recorded at 2 h intervals for the duration of the experiment (48 h). The data were then analysed using an integrated metric: Relative Wound density. For the tube formation assay, 48-well culture plates on ice were incubated with 144 µl of chilled BD Matrigel matrix (10 mg/ml) per well at 37°C for 60 min. hPMEC (6×10^4^) were resuspended in culture medium with the indicated concentration of pMSC-derived exosomes (5, 10 or 20 µg/ml) and incubated for up to 24 h at 37°C. The number of networks formed was determined using the IncuCyte™ system.

### Proliferation Assay

A real-time imaging system (IncuCyteTM) was used to measure cell proliferation using non-label cell monolayer confluence approach. pMSC confluence was measure before and after the treatment (1%, 3% and 8% O_2_, 48 h). IncuCyteTM provide the capability to acquire high quality, phase-contrast images and an integrated confluence metric as a surrogate for cell number [36]. We used similar approach for to determine the effect of pMSC-derived exosomes on hPMEC proliferation during the migration assay.

### Proteomic Analysis of Exosomes by Mass Spectrometry (MS)

Isolated exosomes were solubilised in 8 M urea in 50 mM ammonium bicarbonate, pH 8.5, and reduced with DTT for 1 h. Proteins were then alkylated in 10 mM iodoacetic acid (IAA) for 1 h in the dark. The sample was diluted to 1∶10 with 50 mM ammonium bicarbonate and digested with trypsin (20 µg) at 37°C for 18 h. The samples were desalted by solid phase extraction using a STAGE tip protocol (Stop and go extraction tips for matrix-assisted laser desorption/ionization, nanoelectrospray, and LC/MS sample pretreatment in proteomics). The eluted peptides were dried by centrifugal evaporation to remove acetonitrile and redissolved in Solvent A. The resulting peptide mixture was analysed by Liquid Chromatography (LC)/Mass Spectrometry (MS) LC-MS/MS on a 5600 Triple TOF mass spectrometer (AB Sciex, Framingham, U.S.A.) equipped with an Eksigent Nanoflow binary gradient HPLC system and a nanospray III ion source. Solvent A was 0.1% formic acid in water and solvent B was 0.1% fomic acid in acetonitrile. MS/MS spectra were collected using Information Dependent Acquisition (IDA) using a survey scan (m/z 350–1500) followed by 25 data-dependent product ion scans of the 25 most intense precursor ions. The data were searched using MASCOT and Protein Pilot search engines.

### Functional Analysis of Exosome Proteome

Proteins identified by MS/MS were analyzed by PANTHER (Protein Analysis THrough Evolutionary Relationships; http://www.pantherdb.org). This software allows the prediction of classify proteins (and their genes) in order to facilitate high-throughput analysis. The classified proteins were classified according to their biological process and molecular function. Differentially expressed proteins were analyzed further by bioinformatic pathway analysis (Ingenuity Pathway Analysis [IPA]; Ingenuity Systems, Mountain View, CA; www.ingenuity.com).

### Statistical Analysis

Data are represented as mean ± SEM, with n = 6 different cells culture (*i.e.* biological replicates) of pMSC isolated from first trimester pregnancies and n = 4 different cell cultures (*i.e* biological replicates) of hPMEC isolated from term placenta. Comparisons between two and more groups were performed by means of unpaired Student’s *t*-test and analysis of variance (ANOVA), respectively. If the ANOVA demonstrated a significant interaction between variables, *post hoc* analyses were performed by the multiple-comparison Bonferroni correction test. Statistical significance was defined at least *p<0.05.*

## Results

### Characterization of Exosome from Placental Mesenchymal Stem Cells

**Figure 1:**
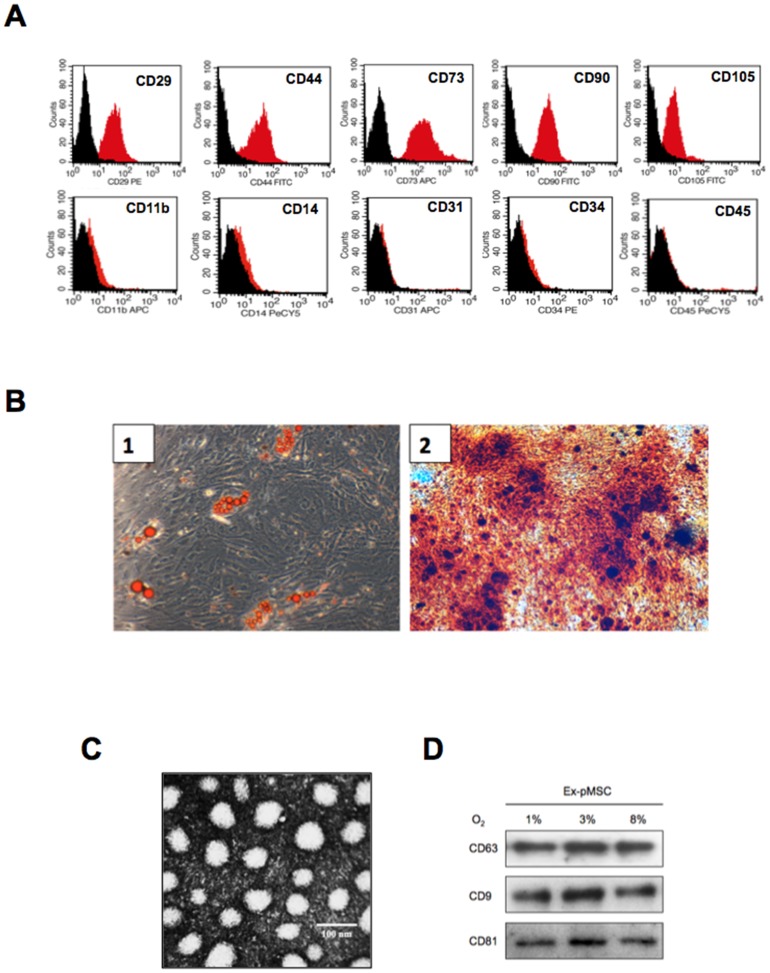
Characterisation of exosomes from placental mesenchymal stem cell (pMSC). Cells were isolated from chorionic villi obtained from first trimester pregnancy and cultured under standard conditions. Exosomes were isolated from pMSC supernatant as was indicated in Methods. (A) Representative flow cytometry histogram of pMSC labeled with positive markers such as CD29, CD44, CD73, CD90 and CD105 (top panel) or negative markers such as CD11b, CD14, CD31, CD34 and CD45 (bottom panel). Black solid peaks represent the isotype controls and the red solid peak represents the marker indicated. (B) Mulit differntiation potential of first trimester placental chorionic villi. 1, Adipogenesis was determined using oil red O staining of lipid droplets after 21 days in adipogenic media. 2, Osteogenesis was determined using alizarin red staining for the mineral matrix deposition after 21 days in osteogenic media. (C) Electron micrograph of exosomes isolated by ultracentrifuge from pMSC. (D) pMSC were exposed to 1%, 3% or 8% O2 during 48 hours and then exosomes proteins were isolated. Samples in each condition were analyzed by western blot after the separation of 20 ug of exosomes protein (same amount of exosome protein lead) for the presence of CD63, CD9 and CD81. In B, *Scale bar* 100 nm.

Cell surface protein expression by pMSC was characterized using flow cytometric analysis. pMSC were labelled with monoclonal antibodies specific for markers indicated in each histogram (Figure 1A). pMSC isolated from first trimester placental villi were positive for CD29^+^, CD44^+^, CD73^+^, CD90^+^, CD105^+^ (top panel) and negative for hematopoietic and endothelial markers: CD11b^-^, CD14^−^, CD31^−^, CD34^−^, CD45^−^ (lower panel). When pMSC were stimulated under adipogenic and osteogenic conditions, they showed characteristic of adipocytes (Figure 1B1; formation of lipid vacuoles) and osteoblast cells (Figure 1B2; red deposits, representing areas of mineralized calcium), respectively. The exosomal particulate fraction isolated from pMSC was examined under transmission electron microscopy. Exosomes were identified as small vesicles between 40–100 nm in a cup-shaped form (Figure 1C). The particulate fraction was further characterized by the expression of specific exosome markers: CD63; CD9; and CD81 by Western blot analysis (Figure 1D).

### Effect of Oxygen Tension on Exosome Release

**Figure 2:**
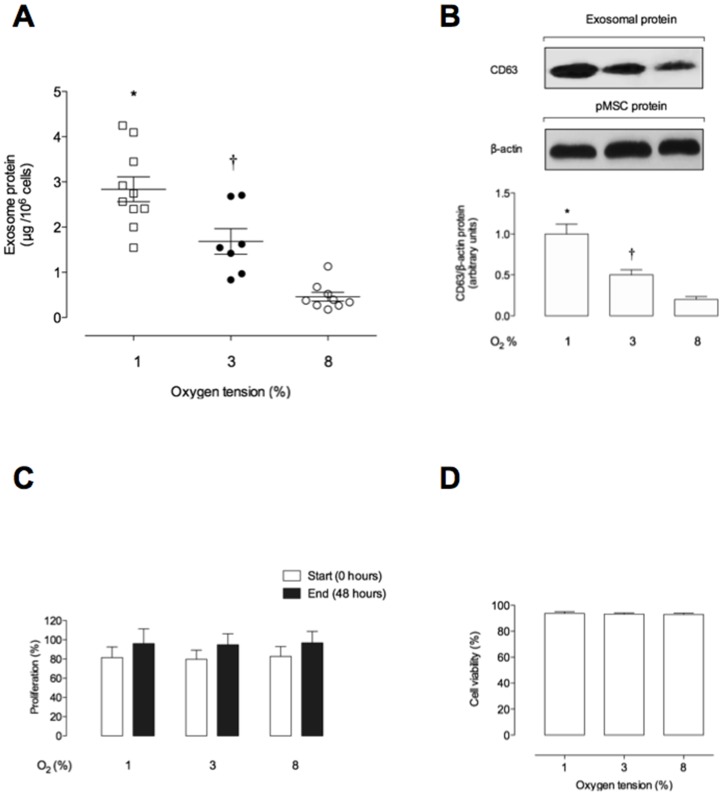
The level of pMSC-derived exosomes compared to low oxygen tension. Exosomes were isolated from pMSC supernatant exposed to 1%, 3% or 8% oxygen per 48 h. (A) Levels of exosomes are presented as protein concentration from 1×10^6^ pMSC cell. (B) Same volume of exosome pellet loaded and analyzed by western blot for CD63 and β-actin in exosome from pMSC and cells, respectively. Lower panel: CD63/β-actin ratio densitometries from data in top panel normalized to 1 in 1% O_2_. (C) Effect of low oxygen tension on pMSC proliferation. (D) Trypan blue dye exclusion test to show residual pMSC cell viability exposed to 1%, 3% or 8% O_2_. Values are mean ± SEM. In A and B, *P<0.001 versus all condition; ^†^ *p*<0.001 versus 8% O_2_.

To determine the effects of oxygen tension on the release of exosomes from pMSC, cells were incubated under atmospheres of 1%, 3% or 8% O_2_ and the exosomes released were quantified (as total exosomal protein µg/10^6^ pMSC). Under these conditions, exosomal protein release averaged 2.8±0.27, 1.6±0.28 and 0.46±0.01 µg protein/10^6^ pMSC, respectively. Exosome release from pMSC was significantly inversely correlated to oxygen tension (ANOVA, *p*<0.001, n = 5; Figure 2A). Furthermore, the relative abundance of the specific exosome marker CD63 in this particulate fraction displayed a similar inverse correlation to oxygen tension, as assessed by Western blot (Figure 2B). During the time course of these experiments, cell proliferation was not significantly affected by oxygen tension (*i.e.* 1%, 3% or 8% O_2_) (Figure 2C). The effect of oxygen tension on exosome release was not associated with a decrease in cell viability (Figure 2D).

### Effect of pMSC-derived Exosomes on Cell Migration

**Figure 3:**
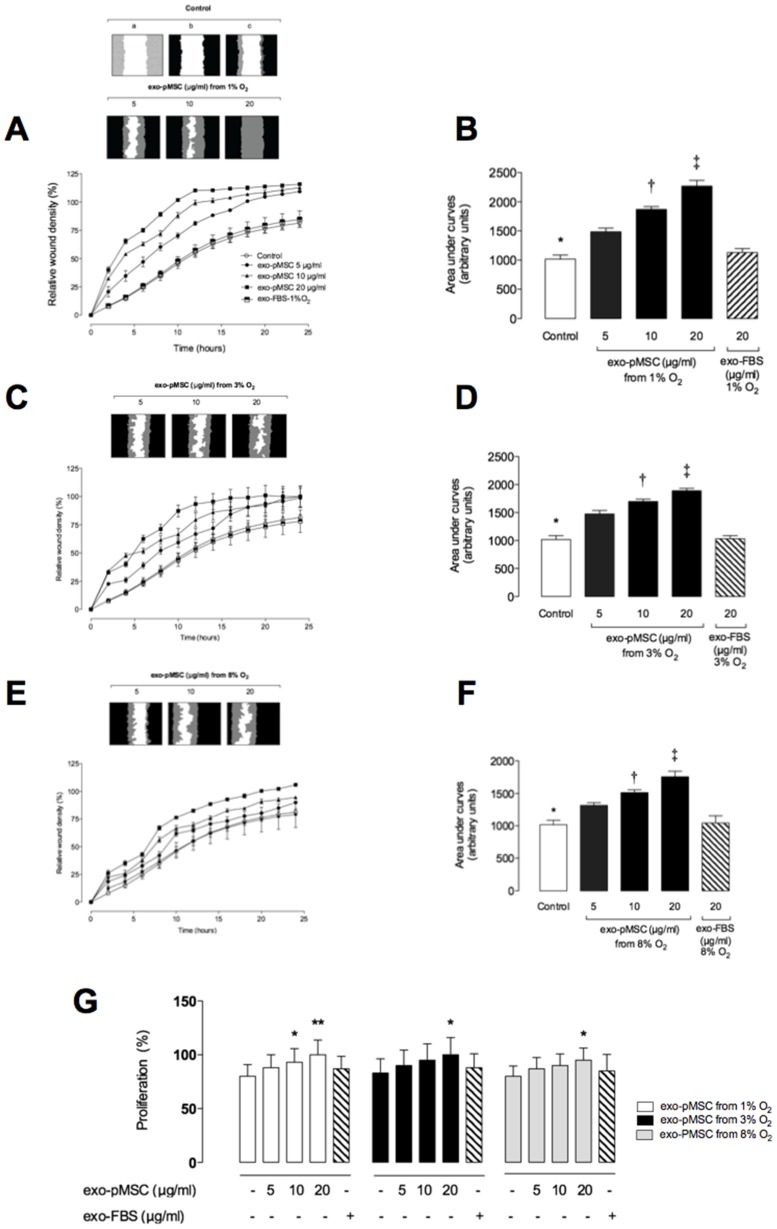
Exosomes increases cell migration in hPMEC. hPMEC were grown to confluence in complete media, wound were made using 96 well WoundMaker and culture in absence (○) or presence (• 5, ▴ 10 or ▪ 20 µg/ml) of exosomal protein obtained from pMSC exposed to different oxygen tension. (A) Top: a, hPMEC image immediately after wounding; b, Graphical representation showing the calculation of initial wound width; c, Graphical representation of cell migration at the midpoint of the experiment. Bottom: The time course of the concentration-dependent effect of exosomal protein from 1% O_2_ on hPMEC, (C) 3% O_2_ or (E) 8% O_2_. (B) Area under curves from data in A, (D) from data in C, (F) from data in E. (G) Effect of pMEC-derived exosomes on hPMEC proliferation. Data represent an n = 6 well each point. Values are mean ± SEM. In B, D and F: *p<0.005 versus all condition; ^†^ *P*<0.005 versus 5 µg/ml; ^‡^ *p*<0.005 versus 10 µg/ml. In G, *p<0.005 versus control (−) with exo-pMSC from 1%, 3% or 8% O_2_; **p<0.001 versus control (−) with exo-pMSC from 1% O_2_.

**Figure 4:**
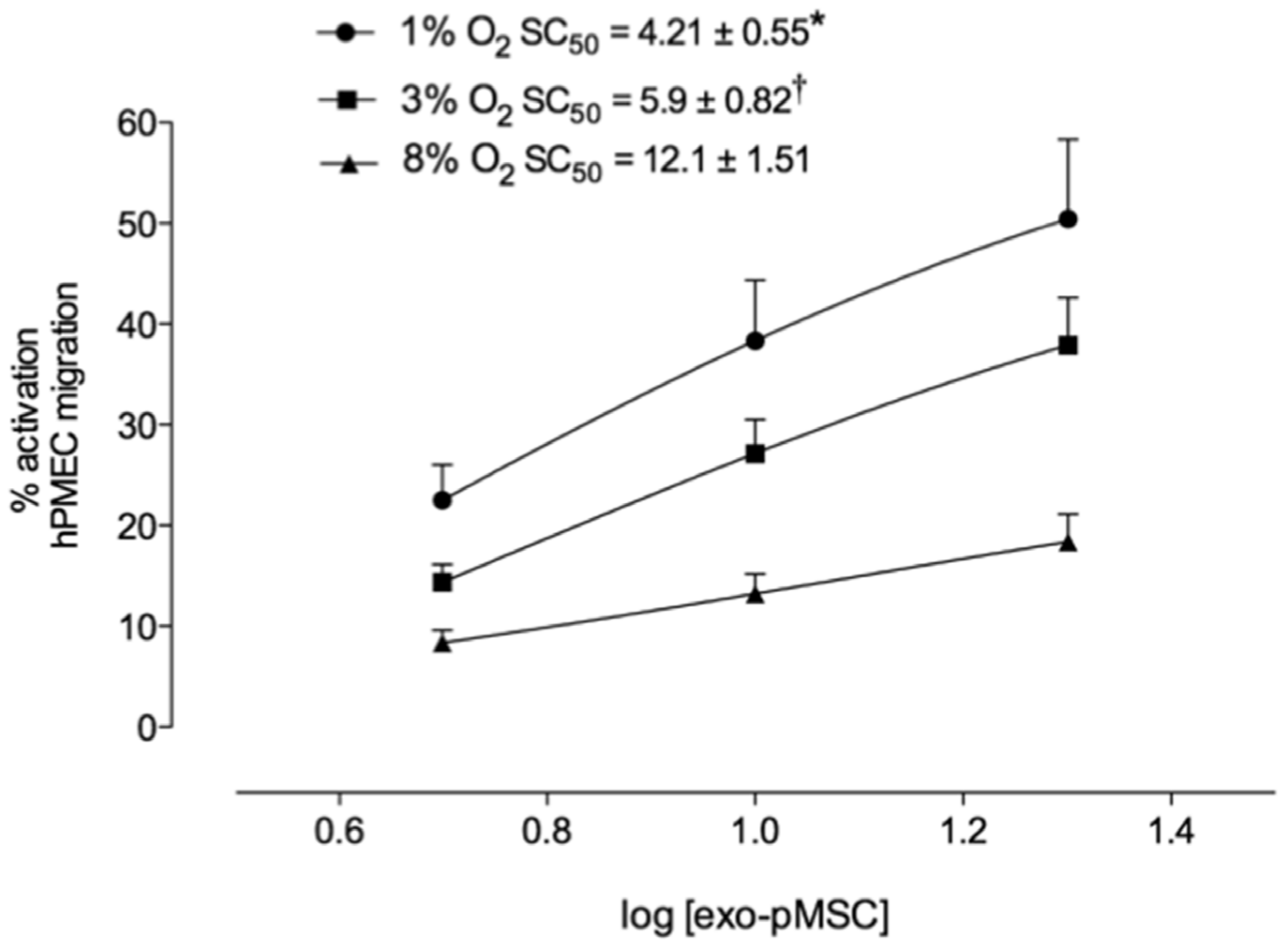
Concentration response of exosomes on hPMEC migration. Activation analysis of exosomes effect on hPMEC migration. Concentration response of exosomal protein from pMSC exposed to 1% (•), 3% (▪) or 8% (▴) O_2_ on hPMEC migration. *Insert*: half-maximal stimulatory concentration (SC_50_) at 6 h. Data represent an n = 6 well each point. Values are mean ± SEM. Insert: *p<0.001 versus all condition; ^†^ *p*<0.005 versus 8% O_2_.

The effects of exosomes (5, 10 or 20 µg protein/ml) isolated from pMSC cultured under 1%, 3% or 8% O_2_ on hPMEC migration are presented in Figure 3 A, C and E. pMSC exosomes significantly increased hPMEC migration in a time- and dose-dependent manner (*p*<0.005, n = 6). In addition, the effect on hPMEC migration was greater when exosomes were prepared from cells cultured under low oxygen tensions. Using the IncuCyte live cell imaging enabled non-invasive system, the cell proliferation based on area metric (confluence) was measurement. Exosomes isolated from pMSC cultures under 1% O_2_ increased significantly the hPMEC proliferation in ∼1.18-fold and ∼1,25-fold with 10 µg/ml and 20 µg/ml, respectively (Figure 4B). Furthermore, exosomes isolated from pMSC cultured under 3% and 8% O2 increased hPMEC proliferation in ∼1.21-fold and ∼1,18-fold using 20 µg/ml, respectively. We did not find significant effect of FBS-derived exosome on hPMEC migration and proliferation. Half-maximal stimulatory time (*ST_50_*) and half-maximal stimulatory concentration (*SC_50_*) values are presented in Table 1.

**Table 1:** Kinetic characteristic of exosome effects on hPMEC migration.

Condition		*Parameter*
	Exosome [µg/ml)	*ST*_50_
Control	–	9.9±0.19
1% O_2_	5	7.9±0.19*
	10	4.0±0.18*
	20	3.9±0.15*^†^
3% O_2_	5	8.0±0.25*
	10	6.2±0.30*
	20	5.9±0.21*
8% O_2_	5	8.2±0.18*
	10	7.8±0.17*
	20	6.4±0.21*

The effect of exosomes isolated from pMSC-conditioned media on hPMEC *in vitro* migration. Data are expressed as half-maximal Stimulatory Time (*ST*_50_ in hours) and represent the mean ± SEM. Primary cultures of endothelial cells were exposed (24 h) to increasing concentration of exosome (0, 5, 10 or 20 µg exosomal protein/ml) obtained from placental mesenchymal stem cell exposed to 1%, 3% or 8% O_2_. hPMEC (CD31^+^) were used in passage 3 for migration assay. *p<0.005 versus control; ^†^ *P*<0.005 versus all condition for *ST*_50_.

Exosome activation was concentration dependent for each condition (half-maximal stimulatory concentration (*SC*_50_) = 4.2±0,5 from 1% O2: versus 5.9±0.6 and 12±1.2 µg/ml from 3% and 8% O_2_, respectively) (Figure 4).

### Effect of pMSC-derived Exosomes on in vitro Tube Formation

**Figure 5:**
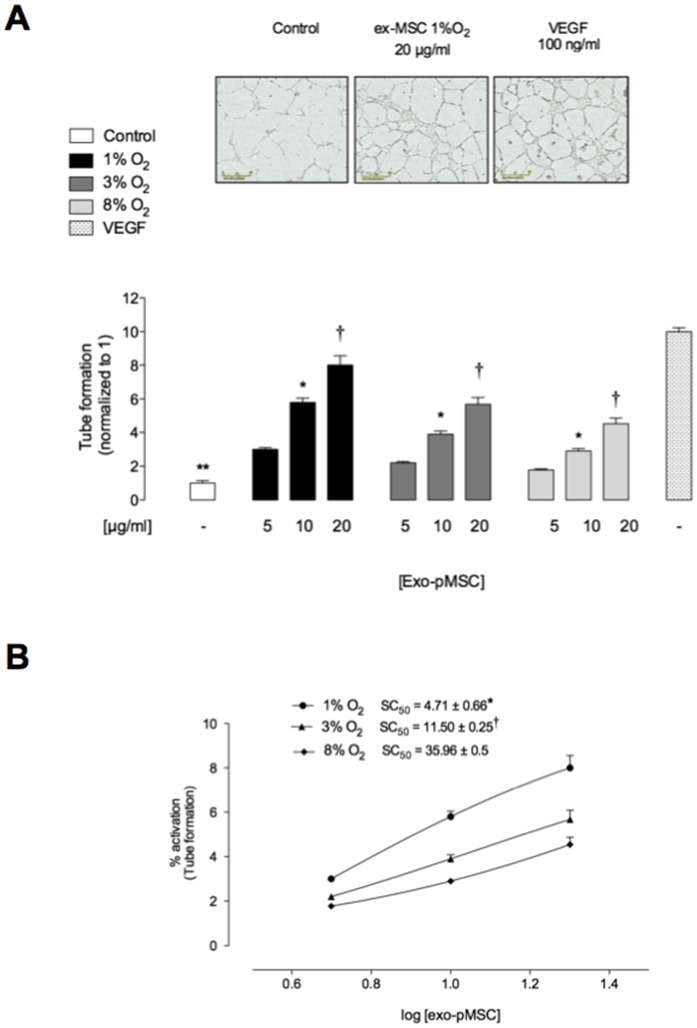
Exosomes from hypoxia increases microvascular tube formation in a dose-dependent manner. hPMEC were incubated in Matrigel in absence or presence of different exosomal protein concentration from pMSC exposed to 1%, 3% or 8% O_2_. (A) Quantitative analysis of the total tube formation. (B) Concentration response from data in A. insert: half-maximal stimulatory concentration (SC_50_) at 16 h. Values are mean ± SEM. In A, **p<0.001 versus all condition; *p<0.005 versus corresponding values in 5 µg/ml, ^†^ *p*<0.005 versus corresponding values in 10 or 5 µg/ml. In B, *P<0.005 versus all values; ^†^ *p*<0.005 versus values in 8% O_2_.

*In vitro* angiogenic tube formation assays were used as a surrogate endpoint to assess the angiogenic effects of pMSC-derived exosomes. pMSC-derived exosomes significantly increased tube formation by hPMEC in a dose- and time-dependent manner when compared to vehicle-treated cells (*p*<0.005, Figure 5A) and inversely correlated to oxygen tension. In addition, exosome-induced tube formation was significantly greater when exosomes were prepared from cells grown under low oxygen tensions (Figure 5A). Half-maximal stimulatory time was 4.71±0.66, 11.50±0.25 and 35.96±0.5 µg/ml for exosomes treatment from pMSC exposed to 1%, 3% and 8% oxygen, respectively.

### Proteomic Analysis of pMSC-derived Exosome

**Figure 6:**
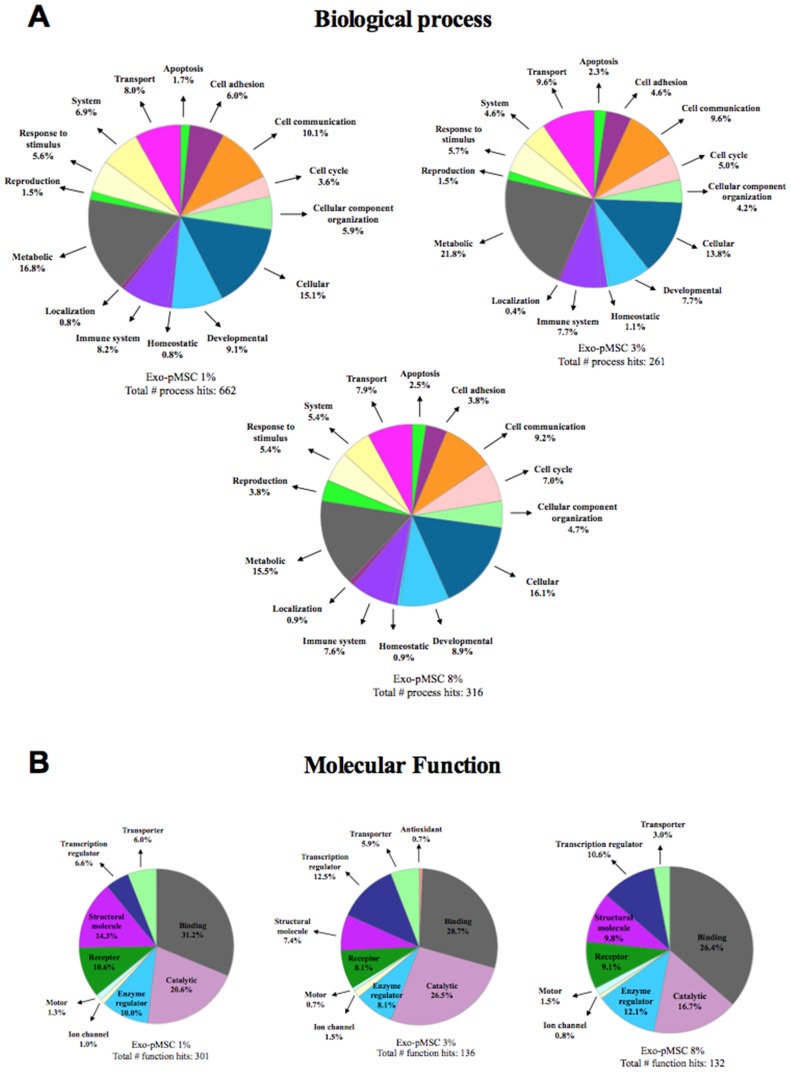
Analysis of pMSC derived-exosomes proteins identified by mass spectrometry using PANTHER software. Exosomal proteins isolated from pMSC exposed to 1%, 3% or 8% O2 were classified using PANTHER program based on their (A) Biological process and (B) Molecular function.

**Figure 7:**
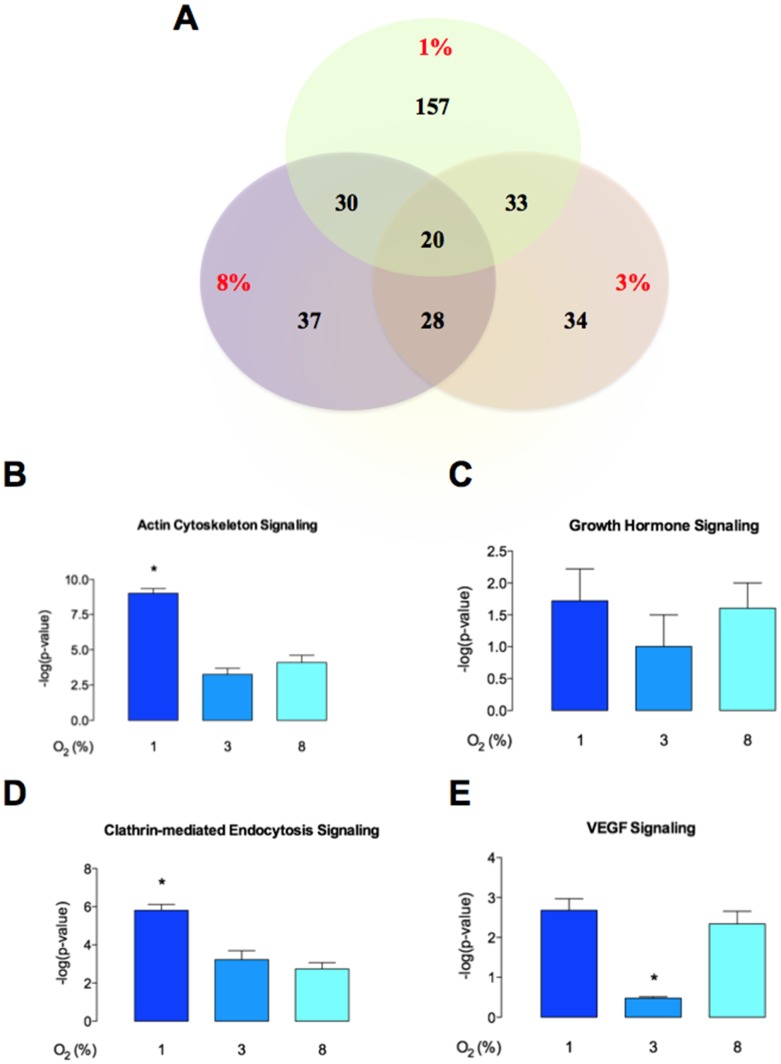
Ingenuity pathway analysis of pMSC derived-exosomes proteins. (A) The Venn diagram depicts the distribution of common and unique proteins identified by nanospray LC-MS/MS (ABSciex 5600) in exosomes released from pMSC exposed to 1%, 3% and 8% oxygen. Comparison of canonical pathways: (B) actin cytoskeleton signaling, (C) growth hormone signaling, (D) VEGF signaling, and (E) clathrin-mediated endocytosis signaling identified by IPA core analysis. Values are mean ± SEM. In B, C, D and E, *p<0.005 versus all condition.

Mass spectrometry analysis identified over 200 exosomal proteins (Table 2). Data were subjected to ontology and pathway analysis using Panther and Gene Ontology algorithms and classified based on biological process and molecular function (Figure 6). In biological process, the most clusters identified were: cellular processes, cell communication, developmental and transport (Figure 6A). In molecular functions, the proteins related to binding and catalytic activity were the greatest recognized (Figure 6B). IPA analysis identified 157 proteins only present in exo-pMSC-1%O_2_ versus 34 and 37 individual proteins present in exo-pMSC-3%O_2_ and exo-pMSC-8%O_2_, respectively_._(Figure 7A). Finally, the canonical pathways associated with our proteins defined by IPA Core analysis and related with cell migration were: actin cytoskeleton signaling, growth hormone signaling, clathrin-mediated endocytosis signaling, and VEGF signaling (Figure 7B-E). Furthermore, canonical pathways were associated with highest protein number in exosomes isolated from pMSC exposed to 1% O_2_ versus 3% and 8% O_2_.

**Table 2:** List of proteins identified in exosomes from pMSC exposed to different oxygen level.

Exo-pMSC-1%O_2_				
ID	Symbol	Entrez Gene Name	Location	Type(s)
A2MG_HUMAN	A2M	alpha-2-macroglobulin	Extracellular Space	transporter
ACTS_HUMAN	ACTA1	actin, alpha 1, skeletal muscle	Cytoplasm	other
ACTB_HUMAN	ACTB	actin, beta	Cytoplasm	other
ACTN1_HUMAN	ACTN1	actinin, alpha 1	Cytoplasm	other
SAHH_HUMAN	AHCY	adenosylhomocysteinase	Cytoplasm	enzyme
FETUA_HUMAN	AHSG	alpha-2-HS-glycoprotein	Extracellular Space	other
AIM1_HUMAN	AIM1	absent in melanoma 1	Extracellular Space	other
ALBU_HUMAN	ALB	albumin	Extracellular Space	transporter
ALDOA_HUMAN	ALDOA	aldolase A, fructose-bisphosphate	Cytoplasm	enzyme
AMOL2_HUMAN	AMOTL2	angiomotin like 2	Plasma Membrane	other
ANXA1_HUMAN	ANXA1	annexin A1	Plasma Membrane	other
ANXA2_HUMAN	ANXA2	annexin A2	Plasma Membrane	other
ANXA5_HUMAN	ANXA5	annexin A5	Plasma Membrane	other
APOA1_HUMAN	APOA1	apolipoprotein A-I	Extracellular Space	transporter
APOB_HUMAN	APOB	apolipoprotein B (including Ag(x) antigen)	Extracellular Space	transporter
APOC3_HUMAN	APOC3	apolipoprotein C-III	Extracellular Space	transporter
APOE_HUMAN	APOE	apolipoprotein E	Extracellular Space	transporter
ARF5_HUMAN	ARF5	ADP-ribosylation factor 5	Cytoplasm	enzyme
ARHG2_HUMAN	ARHGEF2	Rho/Rac guanine nucleotide exchange factor (GEF) 2	Cytoplasm	other
ARPC3_HUMAN	ARPC3	actin related protein 2/3 complex, subunit 3, 21 kDa	Cytoplasm	other
ASB18_HUMAN	ASB18	ankyrin repeat and SOCS box containing 18	unknown	other
ASH1L_HUMAN	ASH1L	ash1 (absent, small, or homeotic)-like (Drosophila)	Nucleus	transcription regulator
A16L1_HUMAN	ATG16L1	autophagy related 16-like 1 (S. cerevisiae)	Cytoplasm	other
AT8B1_HUMAN	ATP8B1	ATPase, aminophospholipid transporter, class I, type 8B, member 1	Plasma Membrane	transporter
ATRN_HUMAN	ATRN	attractin	Extracellular Space	other
B3GN1_HUMAN	B3GNT1	UDP-GlcNAc:betaGal beta-1,3-N-acetylglucosaminyltransferase 1	Cytoplasm	enzyme
BCL3_HUMAN	BCL3	B-cell CLL/lymphoma 3	Nucleus	transcription regulator
BCDO1_HUMAN	BCMO1	beta-carotene 15,15′-monooxygenase 1	Cytoplasm	enzyme
BEND5_HUMAN	BEND5	BEN domain containing 5	Cytoplasm	other
BMP1_HUMAN	BMP1	bone morphogenetic protein 1	Extracellular Space	peptidase
CJ118_HUMAN	C10orf118	chromosome 10 open reading frame 118	unknown	other
C1QT3_HUMAN	C1QTNF3	C1q and tumor necrosis factor related protein 3	Extracellular Space	other
CO3_HUMAN	C3	complement component 3	Extracellular Space	peptidase
CO5_HUMAN	C5	complement component 5	Extracellular Space	cytokine
CI114_HUMAN	C9orf114	chromosome 9 open reading frame 114	Nucleus	other
CALRL_HUMAN	CALCRL	calcitonin receptor-like	Plasma Membrane	G-protein coupled receptor
CAMP2_HUMAN	CAMSAP2	calmodulin regulated spectrin-associated protein family, member 2	unknown	other
CAND1_HUMAN	CAND1	cullin-associated and neddylation-dissociated 1	Cytoplasm	transcription regulator
CC147_HUMAN	CCDC147	coiled-coil domain containing 147	Extracellular Space	other
CB077_HUMAN	CCDC173	coiled-coil domain containing 173	unknown	other
CCD60_HUMAN	CCDC60	coiled-coil domain containing 60	unknown	other
CCD73_HUMAN	CCDC73	coiled-coil domain containing 73	unknown	other
CD44_HUMAN	CD44	CD44 molecule (Indian blood group)	Plasma Membrane	enzyme
CD59_HUMAN	CD59	CD59 molecule, complement regulatory protein	Plasma Membrane	other
CDCA2_HUMAN	CDCA2	cell division cycle associated 2	Nucleus	other
CFAH_HUMAN	CFH	complement factor H	Extracellular Space	other
CGRF1_HUMAN	CGRRF1	cell growth regulator with ring finger domain 1	unknown	other
CLCF1_HUMAN	CLCF1	cardiotrophin-like cytokine factor 1	Extracellular Space	cytokine
CNOT1_HUMAN	CNOT1	CCR4-NOT transcription complex, subunit 1	Cytoplasm	other
COG2_HUMAN	COG2	component of oligomeric golgi complex 2	Cytoplasm	transporter
COCA1_HUMAN	COL12A1	collagen, type XII, alpha 1	Extracellular Space	other
CO1A1_HUMAN	COL1A1	collagen, type I, alpha 1	Extracellular Space	other
CO1A2_HUMAN	COL1A2	collagen, type I, alpha 2	Extracellular Space	other
CO6A1_HUMAN	COL6A1	collagen, type VI, alpha 1	Extracellular Space	other
CO6A2_HUMAN	COL6A2	collagen, type VI, alpha 2	Extracellular Space	other
CO6A3_HUMAN	COL6A3	collagen, type VI, alpha 3	Extracellular Space	other
COMP_HUMAN	COMP	cartilage oligomeric matrix protein	Extracellular Space	other
CBPA1_HUMAN	CPA1	carboxypeptidase A1 (pancreatic)	Extracellular Space	peptidase
SDF1_HUMAN	CXCL12	chemokine (C-X-C motif) ligand 12	Extracellular Space	cytokine
DEN1A_HUMAN	DENND1A	DENN/MADD domain containing 1A	Plasma Membrane	other
MYCPP_HUMAN	DENND4A	DENN/MADD domain containing 4A	Nucleus	other
DYH9_HUMAN	DNAH9	dynein, axonemal, heavy chain 9	Cytoplasm	other
DNJB4_HUMAN	DNAJB4	DnaJ (Hsp40) homolog, subfamily B, member 4	Nucleus	other
DSCAM_HUMAN	DSCAM	Down syndrome cell adhesion molecule	Plasma Membrane	other
EHD3_HUMAN	EHD3	EH-domain containing 3	Cytoplasm	other
EMAL6_HUMAN	EML6	echinoderm microtubule associated protein like 6	unknown	other
ENOA_HUMAN	ENO1	enolase 1, (alpha)	Cytoplasm	transcription regulator
ENTP7_HUMAN	ENTPD7	ectonucleoside triphosphate diphosphohydrolase 7	Cytoplasm	enzyme
HYEP_HUMAN	EPHX1	epoxide hydrolase 1, microsomal (xenobiotic)	Cytoplasm	peptidase
FA10_HUMAN	F10	coagulation factor X	Extracellular Space	peptidase
F13A_HUMAN	F13A1	coagulation factor XIII, A1 polypeptide	Extracellular Space	enzyme
THRB_HUMAN	F2	coagulation factor II (thrombin)	Extracellular Space	peptidase
FA5_HUMAN	F5	coagulation factor V (proaccelerin, labile factor)	Plasma Membrane	enzyme
F117A_HUMAN	FAM117A	family with sequence similarity 117, member A	unknown	transporter
F171B_HUMAN	FAM171B	family with sequence similarity 171, member B	unknown	other
F208B_HUMAN	FAM208B	family with sequence similarity 208, member B	unknown	other
FBLN1_HUMAN	FBLN1	fibulin 1	Extracellular Space	other
FBN1_HUMAN	FBN1	fibrillin 1	Extracellular Space	other
FIBA_HUMAN	FGA	fibrinogen alpha chain	Extracellular Space	other
FGF3_HUMAN	FGF3	fibroblast growth factor 3	Extracellular Space	growth factor
FLNA_HUMAN	FLNA	filamin A, alpha	Cytoplasm	other
FINC_HUMAN	FN1	fibronectin 1	Extracellular Space	enzyme
FRPD3_HUMAN	FRMPD3	FERM and PDZ domain containing 3	unknown	other
GALK1_HUMAN	GALK1	galactokinase 1	Cytoplasm	kinase
G3P_HUMAN	GAPDH	glyceraldehyde-3-phosphate dehydrogenase	Cytoplasm	enzyme
GSCR1_HUMAN	GLTSCR1	glioma tumor suppressor candidate region gene 1	Extracellular Space	other
GBG12_HUMAN	GNG12	guanine nucleotide binding protein (G protein), gamma 12	Plasma Membrane	enzyme
GRIN1_HUMAN	GPRIN1	G protein regulated inducer of neurite outgrowth 1	Plasma Membrane	other
GELS_HUMAN	GSN	gelsolin	Extracellular Space	other
TF2H1_HUMAN	GTF2H1	general transcription factor IIH, polypeptide 1, 62 kDa	Nucleus	transcription regulator
HBB_HUMAN	HBB	hemoglobin, beta	Cytoplasm	transporter
HCN1_HUMAN	HCN1	hyperpolarization activated cyclic nucleotide-gated potassium channel 1	Plasma Membrane	ion channel
HTR5A_HUMAN	HEATR5A	HEAT repeat containing 5A	unknown	other
H13_HUMAN	HIST1H1D	histone cluster 1, H1d	Nucleus	other
H2B1M_HUMAN	HIST1H2BM	histone cluster 1, H2bm	Nucleus	other
H31T_HUMAN	HIST3H3	histone cluster 3, H3	Nucleus	other
HMMR_HUMAN	HMMR	hyaluronan-mediated motility receptor (RHAMM)	Plasma Membrane	other
HS90A_HUMAN	HSP90AA1	heat shock protein 90 kDa alpha (cytosolic), class A member 1	Cytoplasm	enzyme
ENPL_HUMAN	HSP90B1	heat shock protein 90 kDa beta (Grp94), member 1	Cytoplasm	other
GRP78_HUMAN	HSPA5	heat shock 70 kDa protein 5 (glucose-regulated protein, 78 kDa)	Cytoplasm	enzyme
PGBM_HUMAN	HSPG2	heparan sulfate proteoglycan 2	Plasma Membrane	enzyme
HYDIN_HUMAN	HYDIN	HYDIN, axonemal central pair apparatus protein	unknown	other
ALS_HUMAN	IGFALS	insulin-like growth factor binding protein, acid labile subunit	Extracellular Space	other
IGHM_HUMAN	IGHM	immunoglobulin heavy constant mu	Plasma Membrane	transmembrane receptor
IGKC_HUMAN	IGKC	immunoglobulin kappa constant	Extracellular Space	other
IGSF8_HUMAN	IGSF8	immunoglobulin superfamily, member 8	Plasma Membrane	other
IP6K3_HUMAN	IP6K3	inositol hexakisphosphate kinase 3	Cytoplasm	kinase
ITB1_HUMAN	ITGB1	integrin, beta 1 (fibronectin receptor, beta polypeptide, antigen CD29 includes MDF2, MSK12)	Plasma Membrane	transmembrane receptor
ITIH2_HUMAN	ITIH2	inter-alpha-trypsin inhibitor heavy chain 2	Extracellular Space	other
ITIH3_HUMAN	ITIH3	inter-alpha-trypsin inhibitor heavy chain 3	Extracellular Space	other
IRK2_HUMAN	KCNJ2	potassium inwardly-rectifying channel, subfamily J, member 2	Plasma Membrane	ion channel
KI20B_HUMAN	KIF20B	kinesin family member 20B	Nucleus	enzyme
IMB1_HUMAN	KPNB1	karyopherin (importin) beta 1	Nucleus	transporter
K2C1_HUMAN	KRT1	keratin 1	Cytoplasm	other
K1C10_HUMAN	KRT10	keratin 10	Cytoplasm	other
K22E_HUMAN	KRT2	keratin 2	Cytoplasm	other
K1C39_HUMAN	KRT39	keratin 39	Cytoplasm	other
K1C9_HUMAN	KRT9	keratin 9	Cytoplasm	other
LAMB1_HUMAN	LAMB1	laminin, beta 1	Extracellular Space	other
LDB1_HUMAN	LDB1	LIM domain binding 1	Nucleus	transcription regulator
LG3BP_HUMAN	LGALS3BP	lectin, galactoside-binding, soluble, 3 binding protein	Plasma Membrane	transmembrane receptor
LHPL3_HUMAN	LHFPL3	lipoma HMGIC fusion partner-like 3	unknown	other
CQ054_HUMAN	LINC00469	long intergenic non-protein coding RNA 469	unknown	other
YA033_HUMAN	LOC339524	uncharacterized LOC339524	unknown	other
LONM_HUMAN	LONP1	lon peptidase 1, mitochondrial	Cytoplasm	peptidase
LONF2_HUMAN	LONRF2	LON peptidase N-terminal domain and ring finger 2	unknown	other
LPAR6_HUMAN	LPAR6	lysophosphatidic acid receptor 6	Plasma Membrane	G-protein coupled receptor
LRP1_HUMAN	LRP1	low density lipoprotein receptor-related protein 1	Plasma Membrane	transmembrane receptor
TRFL_HUMAN	LTF	lactotransferrin	Extracellular Space	peptidase
LUM_HUMAN	LUM	lumican	Extracellular Space	other
LY75_HUMAN	LY75	lymphocyte antigen 75	Plasma Membrane	other
MACD1_HUMAN	MACROD1	MACRO domain containing 1	Cytoplasm	enzyme
MAP2_HUMAN	MAP2	microtubule-associated protein 2	Cytoplasm	other
MAST3_HUMAN	MAST3	microtubule associated serine/threonine kinase 3	unknown	kinase
MED16_HUMAN	MED16	mediator complex subunit 16	Nucleus	transcription regulator
MFGM_HUMAN	MFGE8	milk fat globule-EGF factor 8 protein	Extracellular Space	other
MKLN1_HUMAN	MKLN1	muskelin 1, intracellular mediator containing kelch motifs	Cytoplasm	other
MOES_HUMAN	MSN	moesin	Plasma Membrane	other
MTERF_HUMAN	MTERF	mitochondrial transcription termination factor	Cytoplasm	transcription regulator
MYH9_HUMAN	MYH9	myosin, heavy chain 9, non-muscle	Cytoplasm	transporter
MYL6_HUMAN	MYL6	myosin, light chain 6, alkali, smooth muscle and non-muscle	Cytoplasm	other
MYLK_HUMAN	MYLK	myosin light chain kinase	Cytoplasm	kinase
MY18B_HUMAN	MYO18B	myosin XVIIIB	Cytoplasm	other
NCTR1_HUMAN	NCR1	natural cytotoxicity triggering receptor 1	Plasma Membrane	transmembrane receptor
NID1_HUMAN	NID1	nidogen 1	Extracellular Space	other
NOL4_HUMAN	NOL4	nucleolar protein 4	Nucleus	other
NOTC3_HUMAN	NOTCH3	notch 3	Plasma Membrane	transcription regulator
NGBR_HUMAN	NUS1	nuclear undecaprenyl pyrophosphate synthase 1 homolog (S. cerevisiae)	Cytoplasm	other
OGG1_HUMAN	OGG1	8-oxoguanine DNA glycosylase	Nucleus	enzyme
O51F1_HUMAN	OR51F1	olfactory receptor, family 51, subfamily F, member 1	Plasma Membrane	G-protein coupled receptor
ORC5_HUMAN	ORC5	origin recognition complex, subunit 5	Nucleus	other
PDIA1_HUMAN	P4HB	prolyl 4-hydroxylase, beta polypeptide	Cytoplasm	enzyme
PDIA3_HUMAN	PDIA3	protein disulfide isomerase family A, member 3	Cytoplasm	peptidase
F261_HUMAN	PFKFB1	6-phosphofructo-2-kinase/fructose-2,6-biphosphatase 1	Cytoplasm	kinase
PGAM1_HUMAN	PGAM1	phosphoglycerate mutase 1 (brain)	Cytoplasm	phosphatase
PI3R4_HUMAN	PIK3R4	phosphoinositide-3-kinase, regulatory subunit 4	Cytoplasm	kinase
KPYM_HUMAN	PKM	pyruvate kinase, muscle	unknown	kinase
PLCL1_HUMAN	PLCL1	phospholipase C-like 1	Cytoplasm	enzyme
PLMN_HUMAN	PLG	plasminogen	Extracellular Space	peptidase
PLPL8_HUMAN	PNPLA8	patatin-like phospholipase domain containing 8	Cytoplasm	enzyme
POSTN_HUMAN	POSTN	periostin, osteoblast specific factor	Extracellular Space	other
P2R3C_HUMAN	PPP2R3C	protein phosphatase 2, regulatory subunit B”, gamma	Cytoplasm	other
PREX1_HUMAN	PREX1	phosphatidylinositol-3,4,5-trisphosphate-dependent Rac exchange factor 1	Cytoplasm	other
PRP31_HUMAN	PRPF31	PRP31 pre-mRNA processing factor 31 homolog (S. cerevisiae)	Nucleus	other
PSA3_HUMAN	PSMA3	proteasome (prosome, macropain) subunit, alpha type, 3	Cytoplasm	peptidase
PSA7L_HUMAN	PSMA8	proteasome (prosome, macropain) subunit, alpha type, 8	Cytoplasm	peptidase
PSB5_HUMAN	PSMB5	proteasome (prosome, macropain) subunit, beta type, 5	Cytoplasm	peptidase
PSB6_HUMAN	PSMB6	proteasome (prosome, macropain) subunit, beta type, 6	Cytoplasm	peptidase
PSB7_HUMAN	PSMB7	proteasome (prosome, macropain) subunit, beta type, 7	Cytoplasm	peptidase
PTX3_HUMAN	PTX3	pentraxin 3, long	Extracellular Space	other
PUSL1_HUMAN	PUSL1	pseudouridylate synthase-like 1	unknown	enzyme
PZP_HUMAN	PZP	pregnancy-zone protein	Extracellular Space	other
ARIP4_HUMAN	RAD54L2	RAD54-like 2 (S. cerevisiae)	Nucleus	transcription regulator
RFX8_HUMAN	RFX8	regulatory factor X, 8	unknown	other
RGPD3_HUMAN	RGPD5 (includes others)	RANBP2-like and GRIP domain containing 5	Nucleus	other
RHPN2_HUMAN	RHPN2	rhophilin, Rho GTPase binding protein 2	Cytoplasm	other
RIMKA_HUMAN	RIMKLA	ribosomal modification protein rimK-like family member A	unknown	other
RN217_HUMAN	RNF217	ring finger protein 217	unknown	enzyme
RL35_HUMAN	RPL35	ribosomal protein L35	Cytoplasm	other
S10AB_HUMAN	S100A11	S100 calcium binding protein A11	Cytoplasm	other
SACS_HUMAN	SACS	spastic ataxia of Charlevoix-Saguenay (sacsin)	Nucleus	other
SALL4_HUMAN	SALL4	sal-like 4 (Drosophila)	Nucleus	other
SDCB1_HUMAN	SDCBP	syndecan binding protein (syntenin)	Plasma Membrane	enzyme
SEM4G_HUMAN	SEMA4G	sema domain, immunoglobulin domain (Ig), transmembrane domain (TM) and short cytoplasmic domain, (semaphorin) 4G	Plasma Membrane	other
SEM6D_HUMAN	SEMA6D	sema domain, transmembrane domain (TM), and cytoplasmic domain, (semaphorin) 6D	Plasma Membrane	other
SPB10_HUMAN	SERPINB10	serpin peptidase inhibitor, clade B (ovalbumin), member 10	Cytoplasm	other
ANT3_HUMAN	SERPINC1	serpin peptidase inhibitor, clade C (antithrombin), member 1	Extracellular Space	other
PEDF_HUMAN	SERPINF1	serpin peptidase inhibitor, clade F (alpha-2 antiplasmin, pigment epithelium derived factor), member 1	Extracellular Space	other
A2AP_HUMAN	SERPINF2	serpin peptidase inhibitor, clade F (alpha-2 antiplasmin, pigment epithelium derived factor), member 2	Extracellular Space	other
SH3L3_HUMAN	SH3BGRL3	SH3 domain binding glutamic acid-rich protein like 3	Nucleus	other
S12A7_HUMAN	SLC12A7	solute carrier family 12 (potassium/chloride transporters), member 7	Plasma Membrane	transporter
S13A4_HUMAN	SLC13A4	solute carrier family 13 (sodium/sulfate symporters), member 4	Plasma Membrane	transporter
SL9C2_HUMAN	SLC9C2	solute carrier family 9, member C2 (putative)	unknown	other
SNTB2_HUMAN	SNTB2	syntrophin, beta 2 (dystrophin-associated protein A1, 59 kDa, basic component 2)	Plasma Membrane	other
SPAT7_HUMAN	SPATA7	spermatogenesis associated 7	unknown	other
STAB2_HUMAN	STAB2	stabilin 2	Plasma Membrane	transmembrane receptor
ST3L1_HUMAN	STAG3L1	stromal antigen 3-like 1	unknown	other
TBL2_HUMAN	TBL2	transducin (beta)-like 2	Plasma Membrane	other
TBPL1_HUMAN	TBPL1	TBP-like 1	Nucleus	transcription regulator
TBX20_HUMAN	TBX20	T-box 20	Nucleus	transcription regulator
TRFE_HUMAN	TF	transferrin	Extracellular Space	transporter
THA11_HUMAN	THAP11	THAP domain containing 11	Nucleus	other
TSP1_HUMAN	THBS1	thrombospondin 1	Extracellular Space	other
THY1_HUMAN	THY1	Thy-1 cell surface antigen	Plasma Membrane	other
TIAR_HUMAN	TIAL1	TIA1 cytotoxic granule-associated RNA binding protein-like 1	Nucleus	transcription regulator
TIMP1_HUMAN	TIMP1	TIMP metallopeptidase inhibitor 1	Extracellular Space	other
TKT_HUMAN	TKT	transketolase	Cytoplasm	enzyme
TLN1_HUMAN	TLN1	talin 1	Plasma Membrane	other
TENA_HUMAN	TNC	tenascin C	Extracellular Space	other
TNAP3_HUMAN	TNFAIP3	tumor necrosis factor, alpha-induced protein 3	Nucleus	enzyme
P53_HUMAN	TP53	tumor protein p53	Nucleus	transcription regulator
TPIS_HUMAN	TPI1	triosephosphate isomerase 1	Cytoplasm	enzyme
TPC12_HUMAN	TRAPPC12	trafficking protein particle complex 12	unknown	other
TITIN_HUMAN	TTN	titin	unknown	kinase
TTYH3_HUMAN	TTYH3	tweety homolog 3 (Drosophila)	Plasma Membrane	ion channel
TBA1B_HUMAN	TUBA1B	tubulin, alpha 1b	Cytoplasm	other
TBB5_HUMAN	TUBB	tubulin, beta class I	Cytoplasm	other
TBB1_HUMAN	TUBB1	tubulin, beta 1 class VI	Cytoplasm	other
TBB2A_HUMAN	TUBB2A	tubulin, beta 2A class IIa	Cytoplasm	other
TYK2_HUMAN	TYK2	tyrosine kinase 2	Plasma Membrane	kinase
UBQLN_HUMAN	UBQLNL	ubiquilin-like	unknown	other
UD2A3_HUMAN	UGT2A3	UDP glucuronosyltransferase 2 family, polypeptide A3	Plasma Membrane	enzyme
USMG5_HUMAN	USMG5	up-regulated during skeletal muscle growth 5 homolog (mouse)	Cytoplasm	other
VAT1_HUMAN	VAT1	vesicle amine transport protein 1 homolog (T. californica)	Plasma Membrane	transporter
CSPG2_HUMAN	VCAN	versican	Extracellular Space	other
VIME_HUMAN	VIM	vimentin	Cytoplasm	other
VTNC_HUMAN	VTN	vitronectin	Extracellular Space	other
WWC2_HUMAN	WWC2	WW and C2 domain containing 2	unknown	other
1433E_HUMAN	YWHAE	tyrosine 3-monooxygenase/tryptophan 5-monooxygenase activation protein, epsilon polypeptide	Cytoplasm	other
ZN268_HUMAN	ZNF268	zinc finger protein 268	Nucleus	other
ZN510_HUMAN	ZNF510	zinc finger protein 510	Nucleus	other
ZN516_HUMAN	ZNF516	zinc finger protein 516	Nucleus	other
ZN599_HUMAN	ZNF599	zinc finger protein 599	unknown	other
ZN729_HUMAN	ZNF729	zinc finger protein 729	unknown	other
ZNF74_HUMAN	ZNF74	zinc finger protein 74	Nucleus	other
**Exo-pMSC-3%O_2_**				
**ID**	**Symbol**	**Entrez Gene Name**	**Location**	**Type(s)**
ABCA1_HUMAN	ABCA1	ATP-binding cassette, sub-family A (ABC1), member 1	Plasma Membrane	transporter
MRP1_HUMAN	ABCC1	ATP-binding cassette, sub-family C (CFTR/MRP), member 1	Plasma Membrane	transporter
ACTB_HUMAN	ACTB	actin, beta	Cytoplasm	other
ADCK4_HUMAN	ADCK4	aarF domain containing kinase 4	Cytoplasm	kinase
FETA_HUMAN	AFP	alpha-fetoprotein	Extracellular Space	transporter
FETUA_HUMAN	AHSG	alpha-2-HS-glycoprotein	Extracellular Space	other
ALBU_HUMAN	ALB	albumin	Extracellular Space	transporter
ARHG2_HUMAN	ARHGEF2	Rho/Rac guanine nucleotide exchange factor (GEF) 2	Cytoplasm	other
BMR1B_HUMAN	BMPR1B	bone morphogenetic protein receptor, type IB	Plasma Membrane	kinase
BPTF_HUMAN	BPTF	bromodomain PHD finger transcription factor	Nucleus	transcription regulator
CJ118_HUMAN	C10orf118	chromosome 10 open reading frame 118	unknown	other
ERG28_HUMAN	C14orf1	chromosome 14 open reading frame 1	Cytoplasm	other
CH073_HUMAN	C8orf73	chromosome 8 open reading frame 73	unknown	other
CCD80_HUMAN	CCDC80	coiled-coil domain containing 80	Nucleus	other
MPIP1_HUMAN	CDC25A	cell division cycle 25 homolog A (S. pombe)	Nucleus	phosphatase
CDA7L_HUMAN	CDCA7L	cell division cycle associated 7-like	Nucleus	other
CDK13_HUMAN	CDK13	cyclin-dependent kinase 13	Nucleus	kinase
CNGA1_HUMAN	CNGA1	cyclic nucleotide gated channel alpha 1	Plasma Membrane	ion channel
COG2_HUMAN	COG2	component of oligomeric golgi complex 2	Cytoplasm	transporter
CODA1_HUMAN	COL13A1	collagen, type XIII, alpha 1	Plasma Membrane	other
CO1A1_HUMAN	COL1A1	collagen, type I, alpha 1	Extracellular Space	other
DIAC_HUMAN	CTBS	chitobiase, di-N-acetyl-	Cytoplasm	enzyme
DUPD1_HUMAN	DUPD1	dual specificity phosphatase and pro isomerase domain containing 1	unknown	enzyme
RNZ2_HUMAN	ELAC2	elaC homolog 2 (E. coli)	Nucleus	enzyme
EPC2_HUMAN	EPC2	enhancer of polycomb homolog 2 (Drosophila)	unknown	other
XPF_HUMAN	ERCC4	excision repair cross-complementing rodent repair deficiency, complementation group 4	Nucleus	enzyme
ERI2_HUMAN	ERI2	ERI1 exoribonuclease family member 2	unknown	other
ETS1_HUMAN	ETS1	v-ets erythroblastosis virus E26 oncogene homolog 1 (avian)	Nucleus	transcription regulator
EXTL2_HUMAN	EXTL2	exostoses (multiple)-like 2	Cytoplasm	enzyme
FA10_HUMAN	F10	coagulation factor X	Extracellular Space	peptidase
THRB_HUMAN	F2	coagulation factor II (thrombin)	Extracellular Space	peptidase
F117A_HUMAN	FAM117A	family with sequence similarity 117, member A	unknown	transporter
F168A_HUMAN	FAM168A	family with sequence similarity 168, member A	unknown	other
F208A_HUMAN	FAM208A	family with sequence similarity 208, member A	unknown	other
F210A_HUMAN	FAM210A	family with sequence similarity 210, member A	Cytoplasm	other
FBN1_HUMAN	FBN1	fibrillin 1	Extracellular Space	other
FGF18_HUMAN	FGF18	fibroblast growth factor 18	Extracellular Space	growth factor
FINC_HUMAN	FN1	fibronectin 1	Extracellular Space	enzyme
FNIP2_HUMAN	FNIP2	folliculin interacting protein 2	Cytoplasm	other
VTDB_HUMAN	GC	group-specific component (vitamin D binding protein)	Extracellular Space	transporter
GSCR1_HUMAN	GLTSCR1	glioma tumor suppressor candidate region gene 1	Extracellular Space	other
GOG8A_HUMAN	GOLGA8A/GOLGA8B	golgin A8 family, member B	Cytoplasm	other
GRIN1_HUMAN	GPRIN1	G protein regulated inducer of neurite outgrowth 1	Plasma Membrane	other
HBB_HUMAN	HBB	hemoglobin, beta	Cytoplasm	transporter
HELZ_HUMAN	HELZ	helicase with zinc finger	Nucleus	enzyme
HJURP_HUMAN	HJURP	Holliday junction recognition protein	Nucleus	other
1B39_HUMAN	HLA-B	major histocompatibility complex, class I, B	Plasma Membrane	transmembrane receptor
H90B3_HUMAN	HSP90AB3P	heat shock protein 90 kDa alpha (cytosolic), class B member 3, pseudogene	unknown	other
I22R1_HUMAN	IL22RA1	interleukin 22 receptor, alpha 1	Plasma Membrane	transmembrane receptor
ITIH2_HUMAN	ITIH2	inter-alpha-trypsin inhibitor heavy chain 2	Extracellular Space	other
K2C1_HUMAN	KRT1	keratin 1	Cytoplasm	other
K2C5_HUMAN	KRT5	keratin 5	Cytoplasm	other
AMPL_HUMAN	LAP3	leucine aminopeptidase 3	Cytoplasm	peptidase
TRFL_HUMAN	LTF	lactotransferrin	Extracellular Space	peptidase
MLAS1_HUMAN	MLLT4-AS1	MLLT4 antisense RNA 1	unknown	other
MYH4_HUMAN	MYH4	myosin, heavy chain 4, skeletal muscle	Cytoplasm	enzyme
ULA1_HUMAN	NAE1	NEDD8 activating enzyme E1 subunit 1	Cytoplasm	enzyme
NGEF_HUMAN	NGEF	neuronal guanine nucleotide exchange factor	Cytoplasm	other
NAL13_HUMAN	NLRP13	NLR family, pyrin domain containing 13	unknown	other
NOL4_HUMAN	NOL4	nucleolar protein 4	Nucleus	other
NTM1A_HUMAN	NTMT1	N-terminal Xaa-Pro-Lys N-methyltransferase 1	Nucleus	enzyme
TEN3_HUMAN	ODZ3	odz, odd Oz/ten-m homolog 3 (Drosophila)	Plasma Membrane	other
O10A7_HUMAN	OR10A7	olfactory receptor, family 10, subfamily A, member 7	Plasma Membrane	other
OSGEP_HUMAN	OSGEP	O-sialoglycoprotein endopeptidase	unknown	peptidase
PGK1_HUMAN	PGK1	phosphoglycerate kinase 1	Cytoplasm	kinase
PHF10_HUMAN	PHF10	PHD finger protein 10	Nucleus	other
KCC1B_HUMAN	PNCK	pregnancy up-regulated non-ubiquitously expressed CaM kinase	unknown	kinase
P2R3C_HUMAN	PPP2R3C	protein phosphatase 2, regulatory subunit B”, gamma	Cytoplasm	other
PR38A_HUMAN	PRPF38A	PRP38 pre-mRNA processing factor 38 (yeast) domain containing A	Nucleus	other
PTPRK_HUMAN	PTPRK	protein tyrosine phosphatase, receptor type, K	Plasma Membrane	phosphatase
PUSL1_HUMAN	PUSL1	pseudouridylate synthase-like 1	unknown	enzyme
PXDN_HUMAN	PXDN	peroxidasin homolog (Drosophila)	Extracellular Space	enzyme
PXK_HUMAN	PXK	PX domain containing serine/threonine kinase	Cytoplasm	kinase
PZP_HUMAN	PZP	pregnancy-zone protein	Extracellular Space	other
RAB10_HUMAN	RAB10	RAB10, member RAS oncogene family	Cytoplasm	enzyme
REST_HUMAN	REST	RE1-silencing transcription factor	Nucleus	transcription regulator
RFX8_HUMAN	RFX8	regulatory factor X, 8	unknown	other
SALL4_HUMAN	SALL4	sal-like 4 (Drosophila)	Nucleus	other
A2AP_HUMAN	SERPINF2	serpin peptidase inhibitor, clade F (alpha-2 antiplasmin, pigment epithelium derived factor), member 2	Extracellular Space	other
SHSA7_HUMAN	SHISA7	shisa homolog 7 (Xenopus laevis)	unknown	other
S12A7_HUMAN	SLC12A7	solute carrier family 12 (potassium/chloride transporters), member 7	Plasma Membrane	transporter
S35A1_HUMAN	SLC35A1	solute carrier family 35 (CMP-sialic acid transporter), member A1	Cytoplasm	transporter
SMTN_HUMAN	SMTN	smoothelin	Extracellular Space	other
SPC25_HUMAN	SPC25	SPC25, NDC80 kinetochore complex component, homolog (S. cerevisiae)	Cytoplasm	other
SPP24_HUMAN	SPP2	secreted phosphoprotein 2, 24 kDa	Extracellular Space	other
SYNJ1_HUMAN	SYNJ1	synaptojanin 1	Cytoplasm	phosphatase
TANC1_HUMAN	TANC1	tetratricopeptide repeat, ankyrin repeat and coiled-coil containing 1	Plasma Membrane	other
TCEA3_HUMAN	TCEA3	transcription elongation factor A (SII), 3	Nucleus	transcription regulator
TET1_HUMAN	TET1	tet methylcytosine dioxygenase 1	Nucleus	other
TEX2_HUMAN	TEX2	testis expressed 2	unknown	other
TRFE_HUMAN	TF	transferrin	Extracellular Space	transporter
TGFR1_HUMAN	TGFBR1	transforming growth factor, beta receptor 1	Plasma Membrane	kinase
TSP1_HUMAN	THBS1	thrombospondin 1	Extracellular Space	other
TITIN_HUMAN	TTN	titin	unknown	kinase
VAT1_HUMAN	VAT1	vesicle amine transport protein 1 homolog (T. californica)	Plasma Membrane	transporter
MELT_HUMAN	VEPH1	ventricular zone expressed PH domain homolog 1 (zebrafish)	Nucleus	other
VTNC_HUMAN	VTN	vitronectin	Extracellular Space	other
XKR3_HUMAN	XKR3	XK, Kell blood group complex subunit-related family, member 3	unknown	other
XPO5_HUMAN	XPO5	exportin 5	Nucleus	transporter
ZDH19_HUMAN	ZDHHC19	zinc finger, DHHC-type containing 19	unknown	other
ZMYM4_HUMAN	ZMYM4	zinc finger, MYM-type 4	unknown	other
ZN143_HUMAN	ZNF143	zinc finger protein 143	Nucleus	transcription regulator
ZN333_HUMAN	ZNF333	zinc finger protein 333	Nucleus	other
ZN486_HUMAN	ZNF486	zinc finger protein 486	Nucleus	other
ZN516_HUMAN	ZNF516	zinc finger protein 516	Nucleus	other
ZN607_HUMAN	ZNF607	zinc finger protein 607	Nucleus	other
ZN645_HUMAN	ZNF645	zinc finger protein 645	Extracellular Space	other
ZN646_HUMAN	ZNF646	zinc finger protein 646	Nucleus	other
ZN770_HUMAN	ZNF770	zinc finger protein 770	unknown	other
ZN808_HUMAN	ZNF808	zinc finger protein 808	unknown	other
ZN865_HUMAN	ZNF865	zinc finger protein 865	unknown	other
ZNF98_HUMAN	ZNF98	zinc finger protein 98	unknown	other
**Exo-pMSC-8%O_2_**				
**ID**	**Symbol**	**Entrez Gene Name**	**Location**	**Type(s)**
ACTS_HUMAN	ACTA1	actin, alpha 1, skeletal muscle	Cytoplasm	other
ACTB_HUMAN	ACTB	actin, beta	Cytoplasm	other
PACA_HUMAN	ADCYAP1	adenylate cyclase activating polypeptide 1 (pituitary)	Extracellular Space	other
FETA_HUMAN	AFP	alpha-fetoprotein	Extracellular Space	transporter
FETUA_HUMAN	AHSG	alpha-2-HS-glycoprotein	Extracellular Space	other
ALBU_HUMAN	ALB	albumin	Extracellular Space	transporter
ANKH1_HUMAN	ANKHD1	ankyrin repeat and KH domain containing 1	unknown	other
ARMX1_HUMAN	ARMCX1	armadillo repeat containing, X-linked 1	unknown	other
ASXL3_HUMAN	ASXL3	additional sex combs like 3 (Drosophila)	unknown	other
ATG2B_HUMAN	ATG2B	autophagy related 2B	unknown	other
BAI1_HUMAN	BAI1	brain-specific angiogenesis inhibitor 1	Plasma Membrane	G-protein coupled receptor
BCL3_HUMAN	BCL3	B-cell CLL/lymphoma 3	Nucleus	transcription regulator
CL043_HUMAN	C12orf43	chromosome 12 open reading frame 43	unknown	other
CO3_HUMAN	C3	complement component 3	Extracellular Space	peptidase
CALB1_HUMAN	CALB1	calbindin 1, 28 kDa	Cytoplasm	other
CALR_HUMAN	CALR	calreticulin	Cytoplasm	transcription regulator
CAND1_HUMAN	CAND1	cullin-associated and neddylation-dissociated 1	Cytoplasm	transcription regulator
CAD20_HUMAN	CDH20	cadherin 20, type 2	Plasma Membrane	other
CDK4_HUMAN	CDK4	cyclin-dependent kinase 4	Nucleus	kinase
CEP97_HUMAN	CEP97	centrosomal protein 97 kDa	Cytoplasm	other
CIZ1_HUMAN	CIZ1	CDKN1A interacting zinc finger protein 1	Nucleus	transporter
CMBL_HUMAN	CMBL	carboxymethylenebutenolidase homolog (Pseudomonas)	unknown	enzyme
CO1A1_HUMAN	COL1A1	collagen, type I, alpha 1	Extracellular Space	other
CO1A2_HUMAN	COL1A2	collagen, type I, alpha 2	Extracellular Space	other
CSF2R_HUMAN	CSF2RA	colony stimulating factor 2 receptor, alpha, low-affinity (granulocyte-macrophage)	Plasma Membrane	transmembrane receptor
CSTF3_HUMAN	CSTF3	cleavage stimulation factor, 3′ pre-RNA, subunit 3, 77 kDa	Nucleus	other
DIAC_HUMAN	CTBS	chitobiase, di-N-acetyl-	Cytoplasm	enzyme
DG2L6_HUMAN	DGAT2L6	diacylglycerol O-acyltransferase 2-like 6	unknown	other
DYH9_HUMAN	DNAH9	dynein, axonemal, heavy chain 9	Cytoplasm	other
EMAL5_HUMAN	EML5	echinoderm microtubule associated protein like 5	unknown	other
ENPP3_HUMAN	ENPP3	ectonucleotide pyrophosphatase/phosphodiesterase 3	Plasma Membrane	enzyme
FA10_HUMAN	F10	coagulation factor X	Extracellular Space	peptidase
THRB_HUMAN	F2	coagulation factor II (thrombin)	Extracellular Space	peptidase
FA73B_HUMAN	FAM73B	family with sequence similarity 73, member B	unknown	other
FBN1_HUMAN	FBN1	fibrillin 1	Extracellular Space	other
FLT3_HUMAN	FLT3	fms-related tyrosine kinase 3	Plasma Membrane	kinase
FINC_HUMAN	FN1	fibronectin 1	Extracellular Space	enzyme
GLBL3_HUMAN	GLB1L3	galactosidase, beta 1-like 3	unknown	enzyme
GP126_HUMAN	GPR126	G protein-coupled receptor 126	Plasma Membrane	G-protein coupled receptor
GRIN1_HUMAN	GPRIN1	G protein regulated inducer of neurite outgrowth 1	Plasma Membrane	other
HBD_HUMAN	HBD	hemoglobin, delta	Cytoplasm	transporter
HCFC2_HUMAN	HCFC2	host cell factor C2	Nucleus	transcription regulator
IL25_HUMAN	IL25	interleukin 25	Extracellular Space	cytokine
INT4_HUMAN	INTS4	integrator complex subunit 4	Nucleus	other
IQGA1_HUMAN	IQGAP1	IQ motif containing GTPase activating protein 1	Cytoplasm	other
ITA4_HUMAN	ITGA4	integrin, alpha 4 (antigen CD49D, alpha 4 subunit of VLA-4 receptor)	Plasma Membrane	other
ITIH2_HUMAN	ITIH2	inter-alpha-trypsin inhibitor heavy chain 2	Extracellular Space	other
K0232_HUMAN	KIAA0232	KIAA0232	Extracellular Space	other
SKT_HUMAN	KIAA1217	KIAA1217	Cytoplasm	other
KNG1_HUMAN	KNG1	kininogen 1	Extracellular Space	other
K2C1_HUMAN	KRT1	keratin 1	Cytoplasm	other
K1C10_HUMAN	KRT10	keratin 10	Cytoplasm	other
LMBL3_HUMAN	L3MBTL3	l(3)mbt-like 3 (Drosophila)	Nucleus	other
LPHN2_HUMAN	LPHN2	latrophilin 2	Plasma Membrane	G-protein coupled receptor
LRRC9_HUMAN	LRRC9	leucine rich repeat containing 9	unknown	other
TRFL_HUMAN	LTF	lactotransferrin	Extracellular Space	peptidase
MACF1_HUMAN	MACF1	microtubule-actin crosslinking factor 1	Cytoplasm	enzyme
MCLN2_HUMAN	MCOLN2	mucolipin 2	Plasma Membrane	ion channel
M4A10_HUMAN	MS4A10	membrane-spanning 4-domains, subfamily A, member 10	unknown	other
MYB_HUMAN	MYB	v-myb myeloblastosis viral oncogene homolog (avian)	Nucleus	transcription regulator
ULA1_HUMAN	NAE1	NEDD8 activating enzyme E1 subunit 1	Cytoplasm	enzyme
NFL_HUMAN	NEFL	neurofilament, light polypeptide	Cytoplasm	other
NOL4_HUMAN	NOL4	nucleolar protein 4	Nucleus	other
NTF3_HUMAN	NTF3	neurotrophin 3	Extracellular Space	growth factor
O10A7_HUMAN	OR10A7	olfactory receptor, family 10, subfamily A, member 7	Plasma Membrane	other
ORC1_HUMAN	ORC1	origin recognition complex, subunit 1	Nucleus	other
OSBL7_HUMAN	OSBPL7	oxysterol binding protein-like 7	Cytoplasm	other
PARP8_HUMAN	PARP8	poly (ADP-ribose) polymerase family, member 8	unknown	other
PCOC1_HUMAN	PCOLCE	procollagen C-endopeptidase enhancer	Extracellular Space	other
PENK_HUMAN	PENK	proenkephalin	Extracellular Space	other
PHIP_HUMAN	PHIP	pleckstrin homology domain interacting protein	Nucleus	other
PI3R4_HUMAN	PIK3R4	phosphoinositide-3-kinase, regulatory subunit 4	Cytoplasm	kinase
PIWL1_HUMAN	PIWIL1	piwi-like 1 (Drosophila)	Cytoplasm	other
PRDM9_HUMAN	PRDM9	PR domain containing 9	Nucleus	enzyme
PYRD1_HUMAN	PYROXD1	pyridine nucleotide-disulphide oxidoreductase domain 1	unknown	other
PZP_HUMAN	PZP	pregnancy-zone protein	Extracellular Space	other
RBL1_HUMAN	RBL1	retinoblastoma-like 1 (p107)	Nucleus	other
THBG_HUMAN	SERPINA7	serpin peptidase inhibitor, clade A (alpha-1 antiproteinase, antitrypsin), member 7	Extracellular Space	transporter
ANT3_HUMAN	SERPINC1	serpin peptidase inhibitor, clade C (antithrombin), member 1	Extracellular Space	other
SHSA7_HUMAN	SHISA7	shisa homolog 7 (Xenopus laevis)	unknown	other
S12A7_HUMAN	SLC12A7	solute carrier family 12 (potassium/chloride transporters), member 7	Plasma Membrane	transporter
SMC4_HUMAN	SMC4	structural maintenance of chromosomes 4	Nucleus	transporter
RU2B_HUMAN	SNRPB2	small nuclear ribonucleoprotein polypeptide B	Nucleus	other
OSTP_HUMAN	SPP1	secreted phosphoprotein 1	Extracellular Space	cytokine
SPP24_HUMAN	SPP2	secreted phosphoprotein 2, 24 kDa	Extracellular Space	other
F10A1_HUMAN	ST13	suppression of tumorigenicity 13 (colon carcinoma) (Hsp70 interacting protein)	Cytoplasm	other
SPT6H_HUMAN	SUPT6H	suppressor of Ty 6 homolog (S. cerevisiae)	Nucleus	transcription regulator
TRBP2_HUMAN	TARBP2	TAR (HIV-1) RNA binding protein 2	Nucleus	other
TBL3_HUMAN	TBL3	transducin (beta)-like 3	Cytoplasm	peptidase
TET1_HUMAN	TET1	tet methylcytosine dioxygenase 1	Nucleus	other
TRFE_HUMAN	TF	transferrin	Extracellular Space	transporter
TSP1_HUMAN	THBS1	thrombospondin 1	Extracellular Space	other
TM117_HUMAN	TMEM117	transmembrane protein 117	Cytoplasm	other
TMTC3_HUMAN	TMTC3	transmembrane and tetratricopeptide repeat containing 3	unknown	other
TPD53_HUMAN	TPD52L1	tumor protein D52-like 1	Cytoplasm	other
TPM3_HUMAN	TPM3	tropomyosin 3	Cytoplasm	other
TRAF3_HUMAN	TRAF3	TNF receptor-associated factor 3	Cytoplasm	other
UB2V2_HUMAN	UBE2V2	ubiquitin-conjugating enzyme E2 variant 2	Cytoplasm	enzyme
UHRF2_HUMAN	UHRF2	ubiquitin-like with PHD and ring finger domains 2, E3 ubiquitin protein ligase	Nucleus	enzyme
UN13C_HUMAN	UNC13C	unc-13 homolog C (C. elegans)	Cytoplasm	other
VAMP5_HUMAN	VAMP5	vesicle-associated membrane protein 5 (myobrevin)	Plasma Membrane	transporter
VAT1_HUMAN	VAT1	vesicle amine transport protein 1 homolog (T. californica)	Plasma Membrane	transporter
MELT_HUMAN	VEPH1	ventricular zone expressed PH domain homolog 1 (zebrafish)	Nucleus	other
VTNC_HUMAN	VTN	vitronectin	Extracellular Space	other
1433B_HUMAN	YWHAB	tyrosine 3-monooxygenase/tryptophan 5-monooxygenase activation protein, beta polypeptide	Cytoplasm	transcription regulator
ZDH23_HUMAN	ZDHHC23	zinc finger, DHHC-type containing 23	unknown	other
ZMYM3_HUMAN	ZMYM3	zinc finger, MYM-type 3	Nucleus	other
ZN416_HUMAN	ZNF416	zinc finger protein 416	Nucleus	other
ZN671_HUMAN	ZNF671	zinc finger protein 671	Nucleus	other
ZNF74_HUMAN	ZNF74	zinc finger protein 74	Nucleus	other
ZN778_HUMAN	ZNF778	zinc finger protein 778	unknown	other
ZN841_HUMAN	ZNF841	zinc finger protein 841	unknown	other

## Discussion

Mesenchymal stem cells are present in the human placenta during early pregnancy. During early pregnancy, placental vasculogenesis and angiogenesis proceed under low oxygen conditions prior to the establishment of a materno-placental perfusion. The role of MSC in directing and promoting placental vascular development remains to be clearly elucidated. The aim of this study was to establish the effects of oxygen tension on the release of exosomes from pMSC and to determine the effects of pMSC exosomes on endothelial cell migration and tube formation. The data obtained in the study are consistent with the hypothesis that the release of exosomes from pMSC is increased in hypoxic conditions and that pMSC exosomes promote endothelial cell migration and tube formation. Based on the data obtained, we suggest that pMSC exosomes contribute to the development of new vessels and promote angiogenesis within the placenta under low oxygen conditions. During early pregnancy this occurs as a physiological and developmental process. In pathological pregnancies characterized by compromized placental perfusion and ischaemia, such as preeclampsia and intrauterine growth restriction, we propose that pMSC may also increase exosome release as an adaptive response.

Germane to any study seeking to elucidate the physiological or pathophysiological role of exosomes is their specific isolation. Several methods for isolating exosomes have been developed and partially characterized. These methods are primarily based on particle size and density. By definition, exosomes are nanovesicles with a diameter of 30–100 nm, a buoyant density of 1.12 to 1.19 g/ml and express characteristic cell-surface markers. In this study, pMSC exosomes were isolated by differential centrifuge and sucrose gradient purification and were characterized by a diameter of 50 nm, a buoyant density of 1.1270 g/ml, and expressed exosome-specific cell surface markers.

Under hypoxic conditions (1% or 3% O_2_), pMSC exosome release increased by up to 7-fold compared to cells incubated under normoxic conditions (8% O_2_). These data are consistent with the effects of hypoxia on the release of exosomes from umbilical cord (UC)-derived MSCs, where low oxygen tension increases exosome release by ∼ 5.6-fold [37]. Hypoxia also has been reported to increase the release of exosomes from breast cancer cell lines (MCF7, SKBR3, and MDA- MB 231), squamous carcinoma cells (A431 cells) [26] and cardiac myocytes [38]. The mechanism by which hypoxia induces exosome release remains to be clearly established.

Recent evidence suggests that increased release of exosomes from breast cancer cells under hypoxic condition may be mediated by transcriptional factor HIF-1α[39]. In this study, the authors also observed higher expression of miR-210 in exosomes isolated from cancer cells exposed to hypoxia compared to normaxia cell-derived exosomes. Exosomal miR-210 from metastatic cancer cells enhances endothelial cell angiogenesis [40].

In MSCs, HIFs have been reported to promote MSC-mediated angiogenic effect on endothelial cells through the release of interleukin 8, VEGF and other growth factors [41]. It has been demonstrated that the secretion of soluble VEGF requires functional ADP-ribosylation factor 6 (Arf6) [42]. Interestingly, Arf6 is expressed on the membrane of exosomes and may promote exosome release [43]. An association between VEGF and Arf6 within exosomes, however, has not yet been demonstrated. Similarly, HIFs may contribute to the hypoxia-induced release of exosomes from pMSC observed in this study.

Previous studies have established that MSC promote angiogenesis via paracrine mechanisms [44]. The possible contribution of exosomes in mediating such paracrine actions has not been established. It is likely that exosomes were present (and not accounted for) in all conditioned media previously used to establish such paracrine effects. In this study, exosomes were isolated from pMSC, promoting hPMEC cell migration and tube formation. This effect was enhanced when pMSC were cultured under hypoxic conditions. Previously, Zhang *et al.,* 2012, demonstrated that exosomes released from UC-MSC are internalized into umbilical cord endothelial cells and enhance in vitro the proliferation and network formation in a dose-dependent manner [37]. Interestingly, pMSC have ∼3.2-fold higher than that UC-MSC migration capacity [20]. Recently, Mineo *et al*., reported that the effect of exosomes on angiogenesis involves the Src family of kinases [45]. In addition, the role of Src family members in angiogenesis, promotion of tube formation and prevention of their regression has been reported [46], [47]. Recent commentary, suggests that mesenchymal stem cells-derived exosomes may not only afford therapeutic opportunities in regenerative medicine to repair damaged tissue but also in the cell-specific delivery of anticancer agents [48].

The exosomal content is highly dependent on the cell type and pre-conditioning. One of the first exosomal proteomes characterized was from mesothelioma cells, in which 38 different proteins were identified [49]. Studies in cancer cells show the great variability of proteins expressed in exosomes [50]–[54]. Supporting our results, exosomes isolated from a human first trimester cell line (Sw71) Atay *et al.,* using an ion trap mass spectrometry approach, identified proteins implicated in a wide range of cellular processes including: cytoskeleton structure, ion channels, lysosomal degradation, molecular chaperones, amino-acid metabolism, carbohydrate metabolism, lipid metabolism, regulatory proteins, mRNA splicing, immune function and others [55]. Our study provides the first extensive analysis of the proteome of the exosomes derived-MSC primary culture, highlighting the extent of putative functional interactions that may be mediated by exosomes.

Endothelial cell migration requires the initiation of numerous signaling pathways that remodels cytoskeleton. Also, actin and related proteins of cytoskeletal organization are critical for cell motility and migration. From the canonical pathway analysis, we found significantly more proteins associated with actin cytoskeleton, growth hormone, and VEGF signaling in exosomes isolated from pMSC exposed to 1% O_2_ compare to 3% or 8% O_2_. Likewise, clathrin-mediated endocytosis signaling was enhanced, possibly increasing the exosome uptake of target cells., Cell migration, however, is the final functional outcome of multiple pathways and the involvement of other regulatory moieties (*e.g.* miRNA) cannot be negated.

In summary, pMSC isolated from first trimester placenta release exosomes in response to decreased oxygen tension. pMSC exosomes stimulate microvascular endothelial cells migration in a concentration and oxygen-dependent manner, and promote vascular network formation. The data obtained in this study are consistent with the hypothesis that under normal developmental conditions, pMSC promote vasculogenesis and angiogenesis within the early pregnancy placenta via a mechanism(s) involving exosomal trafficking to endothelial cells. We further suggest that in pathological pregnancies associated with under perfusion of the placenta, such as those complicated by pre-eclampsia and intra-uterine growth restriction, increased release of exosomes from pMSC may occur as an adaptive response.
